# End-to-end data-driven weather prediction

**DOI:** 10.1038/s41586-025-08897-0

**Published:** 2025-03-20

**Authors:** Anna Allen, Stratis Markou, Will Tebbutt, James Requeima, Wessel P. Bruinsma, Tom R. Andersson, Michael Herzog, Nicholas D. Lane, Matthew Chantry, J. Scott Hosking, Richard E. Turner

**Affiliations:** 1 Department of Computer Science and Technology, University of Cambridge, Cambridge, UK; 2 Department of Engineering, University of Cambridge, Cambridge, UK; 3 Vector Institute, University of Toronto, Toronto, Ontario Canada; 4 Microsoft Research AI for Science, Cambridge, UK; 5 British Antarctic Survey, Cambridge, UK; 6 Department of Geography, University of Cambridge, Cambridge, UK; 7 European Centre for Medium-Range Weather Forecasts, Reading, UK; 8 The Alan Turing Institute, London, UK; 9 Present Address: The Alan Turing Institute, London, UK; 10 Present Address: Google DeepMind, London, UK

**Keywords:** Environmental sciences, Computer science, Natural hazards, Atmospheric science

## Abstract

Weather prediction is critical for a range of human activities, including transportation, agriculture and industry, as well as for the safety of the general public. Machine learning transforms numerical weather prediction (NWP) by replacing the numerical solver with neural networks, improving the speed and accuracy of the forecasting component of the prediction pipeline^1–6^. However, current models rely on numerical systems at initialization and to produce local forecasts, thereby limiting their achievable gains. Here we show that a single machine learning model can replace the entire NWP pipeline. Aardvark Weather, an end-to-end data-driven weather prediction system, ingests observations and produces global gridded forecasts and local station forecasts. The global forecasts outperform an operational NWP baseline for several variables and lead times. The local station forecasts are skilful for up to ten days of lead time, competing with a post-processed global NWP baseline and a state-of-the-art end-to-end forecasting system with input from human forecasters. End-to-end tuning further improves the accuracy of local forecasts. Our results show that skilful forecasting is possible without relying on NWP at deployment time, which will enable the realization of the full speed and accuracy benefits of data-driven models. We believe that Aardvark Weather will be the starting point for a new generation of end-to-end models that will reduce computational costs by orders of magnitude and enable the rapid, affordable creation of customized models for a range of end users.

## Abstract (web summary)

Aardvark Weather, an end-to-end machine learning model, replaces the entire numerical weather prediction pipeline with a machine learning model, by producing accurate global and local forecasts without relying on numerical solvers, revolutionizing weather prediction with improved speed, accuracy and customization capabilities.

## Main

Numerical weather prediction (NWP) systems are vital for creating weather forecasts required by emergency agencies, transport providers, agriculture, energy providers and the general public. Since the first numerical forecasts were produced in the 1950s, which required 24 h to compute a single-day single-variable forecast on a 700-km grid^7^, NWP systems have undergone a remarkable transformation. Modern systems predict a wide range of variables at lead times of up to 15 days, which is the theoretical limit of medium-range weather forecasting predictability^8^. These systems consist of an intricate series of models of different components of Earth’s atmosphere, building on decades of research in Earth observation, data assimilation, fluid dynamics and statistical post-processing and requiring purpose-built supercomputers to run.

Generating a modern weather forecast begins with the acquisition of observations from a multitude of sources, including remote sensing instruments, in situ observations, radar systems, radiosondes and aircraft data^9^. Some of these data are processed to generate derived products, such as atmospheric motion vectors and surface winds. Raw data and the resulting processed products are fed into a data assimilation system, which combines these with an initial guess from the previous forecast to generate a global approximation of the current state of the atmosphere. This approximation is then used as an initial state for a forecasting system that integrates the equations of fluid mechanics and thermodynamics to output predictions at future lead times. Finally, the resulting predictions from the forecasting system are used for downstream tasks, for example, to generate local forecasts. This step may consist of statistical post-processing and running higher-resolution regional NWP models. Each stage of this pipeline consists of several numerical models chained together, resulting in an intricate workflow^10^ that is challenging to iterate on and improve and requires purpose-built supercomputers to run. This motivates the development of fast, lightweight and customizable alternatives.

With end-to-end machine learning revolutionizing several fields by replacing complex human-designed workflows, it has been suggested that a data-driven model may one day replace the entire NWP pipeline^11^. This will be transformational for weather prediction, reducing computational costs, removing bias from inflexible aspects of NWP systems and enabling fast prototyping and optimization for specific tasks. However, this has not been attempted so far, with studies focusing on applying machine learning to the easiest components of the pipeline. For example, machine learning models have been shown to outperform their operational state-of-the-art counterparts to replace the numerical solver in the forecasting component^1,2,4–6,12^, deriving variables from raw satellite data in pre-processing^13–15^ and post-processing forecast data in the downstream stages^16,17^. Work on replacing the most challenging component, the assimilation system, remains at the stage of developing initial prototypes^3,18–23^. Therefore, the vision of an end-to-end data-driven solution remains aspirational, with conventional NWP systems being essential for all forms of operational forecasting.

In a recent article assessing the prospect of end-to-end deep learning weather prediction, the verdict was that “a number of fundamental breakthroughs are needed before this goal comes into reach”^11^. Here we report that these breakthroughs are happening earlier than expected. We present Aardvark Weather, an end-to-end data-driven weather forecasting system capable of generating predictions with no input from conventional NWP by instead learning a mapping from raw input observations to output forecasts. This allows Aardvark to tackle the complete weather prediction pipeline while being entirely independent from NWP products at prediction time, relying solely on observation data to generate forecasts. We demonstrate that using an order of magnitude fewer observations than those available to operational baselines and orders of magnitude less computational resources, Aardvark is capable of producing forecasts on a global 1.50° grid that achieves lower root mean square error (RMSE) than operational NWP systems across several variables and lead times. Furthermore, we demonstrate that this system provides local forecasts that achieve lower errors than post-processed NWP and a full end-to-end operational forecasting system for several lead times and can be optimized end-to-end to maximize performance over variables and regions of interest.

## Aardvark Weather

**Fig. 1:**
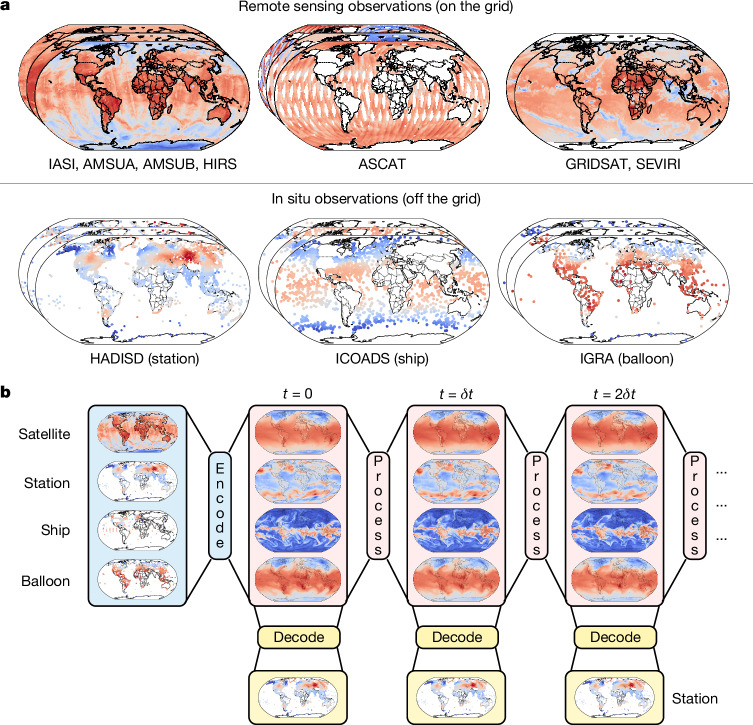
Data and operation of Aardvark Weather. **a**, Different data sources leveraged in Aardvark. The input data consist of observations from remote sensing instruments (top row), which we pre-grid before passing to the model, as well as in situ observations from land and marine observation platforms and radiosondes (bottom row). Each of these data modalities contains several observational variables, of which we selected a subset here for the purposes of illustration. Here we show remote sensing data^40–45^, after performing our gridding step, and raw in situ data^46–48^. Note that the colours in all six plots are meant for illustration purposes. The remote sensing data also include a range of metadata about the measurements, omitted here for simplicity. White areas indicate regions of missing data, which must be handled by the encoder module of Aardvark. **b**, Aardvark at deployment time. First, an encoder module uses raw observations as input to estimate the initial state of the atmosphere across key variables at *t* = 0. Next, a processor module ingests the estimated state to produce a forecast at the next lead time *t* = *δt*. Forecasts at subsequent lead times are produced autoregressively. Finally, a decoder module is applied to the on the grid states to produce off the grid predictions. The modular design of Aardvark allows for pretraining on large high-quality ERA5 reanalysis data^34^. In this figure, the displayed data are the training data used to train each module of Aardvark from the aforementioned sources.

Aardvark Weather is a deep learning model that provides forecasts of eastward wind, northward wind, specific humidity, geopotential and temperature (at 200, 500, 700 and 850 hPa pressure levels), 10-m eastward wind, 10-m northward wind, 2-m temperature and mean sea level pressure on a dense global grid, and station forecasts for 2-m temperature and 10-m wind speed. Aardvark consists of three modules and is designed to leverage high-quality reanalysis data during training while being entirely independent from NWP products at deployment time. Figure 1 (bottom) illustrates the operation of Aardvark, outlining the function of each of its three modules.

First, an encoder module obtains observational data from several sources, both on the grid and off the grid, and produces a gridded initial state. On the grid observations are data modalities on a regular grid, whereas off the grid modalities are available at a set of longitude–latitude locations. To achieve this, we leveraged recent advances from deep learning^24^ in handling off the grid and missing data. This approach to state estimation differs from data assimilation systems used in conventional NWP pipelines. Conventional data assimilation systems use a recurrent update in which the previous forecast is adjusted in light of new observations, similar to Kalman filter recursions in a Markov model. In principle, data assimilation accumulates information from observations across all past time steps. However, in practice, it has been estimated that the effective window size is as short as 4 days (ref. ^25^). Owing to the complexities of training recurrent neural networks, including the need for a spin-up period and gradient instabilities^26^, we opted for a non-recurrent approach.

Once the initial atmospheric state has been estimated, it is used as an input to a processor module, which produces a gridded forecast at a lead time of 24 h. Forecasts at subsequent lead times are produced by autoregressively feeding the predictions of the processor module back to it as an input, similar to the existing approaches in data-driven weather forecasting^1,6^. Finally, task-specific decoder modules ingest these forecasts and produce local predictions. In this study, we considered a decoder designed for a single downstream task, producing local station forecasts. However, this system is suitable for use with several separate decoders for different tasks. Together, the encoder, processor and decoder modules form a neural process^24^, a machine learning system that naturally handles off the grid and missing data. A vision transformer^27^ forms the backbone of the encoder and processor modules, whereas the decoder modules are implemented as a lightweight convolutional architecture. The full set of inputs and outputs for the modules is detailed in Extended Data Table 1.

A key challenge in designing machine learning systems for observational atmospheric data is that the records for many instruments are relatively short, limiting the data available for training. The modular design of Aardvark (Fig. 1) addresses this issue by enabling pretraining using high-fidelity historical reanalysis data before fine-tuning on scarcer observational data. Specifically, we trained the system in a way that mimics how it will be deployed. We started by pretraining the encoder module using raw observations as input and reanalysis data as targets. An advantage of this machine learning approach is that the model can learn to correct for biases in the input observations during training; therefore, no bias correction step was performed on the input data. We also pretrained the processor using reanalysis data for both inputs and targets and then fine-tuned the output of the state-estimation module. In the processor module, the inputs and outputs were both on a regular 1.50° grid to match the reanalysis training data. Next, we trained the decoder using the output of the processor as the input and raw data as targets. This procedure ensures that there is no mismatch between the training and deployment of the system. Finally, we fine-tuned the encoder, processor and decoder modules jointly to optimize the entire model for a specific variable and region. For all modules, we trained on data before 2018 and held out 2018 and 2019 as the test and validation years, respectively.

## Input variables

Accurately estimating the state of the atmosphere requires inputs from various observation sources. Input variables are selected to capture the dynamics both at Earth’s surface and at several levels through the atmosphere. In situ observations are taken from weather stations and ships at surface level and radiosondes at upper levels. As coverage from these instruments is largely confined to the surface, as well as geographically skewed and sparse, remote sensing instruments provide a crucial complementary global data source. Motivated by gains observed in operational NWP systems^28–30^, we selected four primary sources of satellite data: scatterometer data to provide information about surface wind over the ocean, multispectral (approximately ten channels) microwave and infrared sounders, hyperspectral (approximately 10^5^ channels) infrared sounders to provide information on upper-atmosphere temperature and humidity profiles, and geostationary infrared sounder data to provide an instantaneous snapshot of the state of the atmosphere. These observations were made with different time windows ranging from 1 to 24 h before lead time 0. By contrast to operational medium-range NWP systems, observations are only included in the input if they are taken before lead time 0 (ref. ^31^). Figure 1 (top) shows an example of a single time slice of input data to Aardvark for in situ and remote sensing sources, with full details in Extended Data Table 2. These atmospheric observations were augmented by several temporal and orographic variables. Aardvark only ingests approximately 8% of the observations^1^ available to conventional NWP systems^32^, more than an order of magnitude less input data.

## Evaluation of global forecasting

For global gridded forecasts, we compared Aardvark with four baselines. The simplest of these, persistence and hourly climatology, assess whether a forecasting system is skilful. A more challenging comparison is to the two most widely used deterministic operational global NWP systems: the Integrated Forecasting System (IFS) in its high-resolution (HRES) configuration from the European Centre for Medium-Range Weather Forecasts (ECMWF) and the Global Forecast System (GFS) from the National Centers for Environmental Prediction. Although HRES typically outperforms GFS on global metrics, operational centres often use a selection of different models, including GFS, to create their local forecasts; therefore, we included it in our comparison. For each variable, pressure level and lead time, we report the latitude-weighted RMSE, a common metric for assessing the performance of deterministic forecasting systems^33^. For all baselines, we used the ECMWF Reanalysis v5 (ERA5) dataset as the ground truth. This choice was made because this is a standard practice for the evaluation of machine learning NWP models. At present, HRES analysis is of higher quality than ERA5 reanalysis, because ERA5 was developed using cycle Cy41r2 (ref. ^34^), which remained operational until 2017. However, the discrepancies between the two were limited for the test year of 2018.

**Fig. 2:**
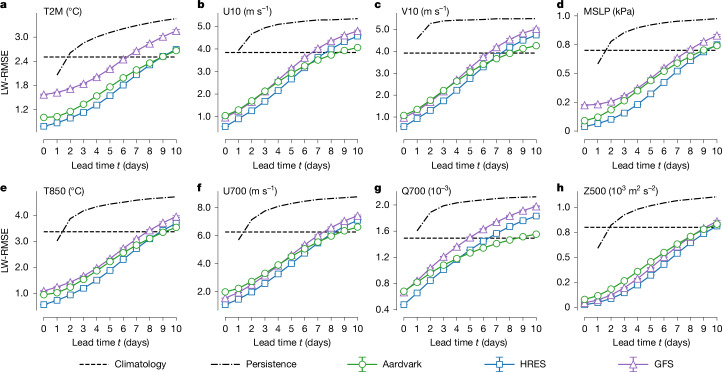
Gridded global forecast performance for selected variables. **a**–**h**, Latitude-weighted RMSE using ERA5 (ref. ^34^) reanalysis data as the ground truth, on the held-out test year (2018), for the four surface variables: 2-m temperature (**a**; T2M), 10-m eastward wind (**b**; U10), 10-m northward wind (**c**; V10) and mean sea level pressure (**d**; MSLP), as well as four headline upper-atmosphere variables: temperature at 850 hPa (**e**; T850), eastward wind at 700 hPa (**f**; U700), specific humidity at 700 hPa (**g**; Q700) and geopotential at 500 hPa (**h**; Z500) as a function of lead time *t*. At lead time *t* = 0, Aardvark predicted the initial atmospheric state from observational data alone. The error at *t* = 0 corresponds to the error in the initial state. Note that HRES has a non-zero error at *t* = 0 compared to ERA5 reanalysis. The HRES forecasts^33^ we used have been conservatively re-gridded to prevent aliasing, and we performed the same operation on the GFS forecasts^49^. We report the mean performance of each system together with 98% confidence intervals in our estimate of the mean performance.

Figure 2 shows the latitude-weighted RMSE performance compared with the baselines for eight headline variables. Here Aardvark matched or outperformed GFS across most lead times, with the only exception being the geopotential at 500 hPa. In addition, for most variables, Aardvark approached the performance of HRES. Overall, Aardvark’s errors were larger at higher atmospheric levels and shorter lead times than those of the operational baselines. This was possibly caused by the higher concentration of observations close to the surface. For longer lead times, a by-product of fine-tuning to minimize errors at future lead times (Methods) is that forecasts tend to become spectrally blurred. This phenomenon is commonly observed in data-driven weather forecasting systems^1,6,35^. A full display of the latitude-weighted RMSE of Aardvark across all variables and levels can be found in Supplementary Fig. 1. Further insights can be drawn from inspecting the power spectra, anomaly correlation coefficients and activities of Aardvark’s forecasts, as shown in Supplementary Figs. 2–4. This analysis suggests that although forecast blurring plays a role, Aardvark produces skilful forecasts and maintains meaningful signals, even at longer lead times.

Figure 3 shows an example of gridded global predictions at lead times of 0, 1, 2 and 4 days for 10-m eastward wind. Aardvark successfully captured the large-scale features of the atmospheric state, both in the mid-latitudes and the tropics. Many details are well represented; for example, the formation of a tropical cyclone in the Southern Indian Ocean closely matched that in the ERA5 reanalysis data. This example hints at the potential of Aardvark for forecasting mesoscale high-impact weather events. Although some spectral blurring of the higher spatial frequencies is evident, these results are of remarkably high fidelity given the limited resolution and range of observations provided to the model. A comprehensive set of spatial plots across all variables is provided in Supplementary Figs. 6–29.

**Fig. 3:**
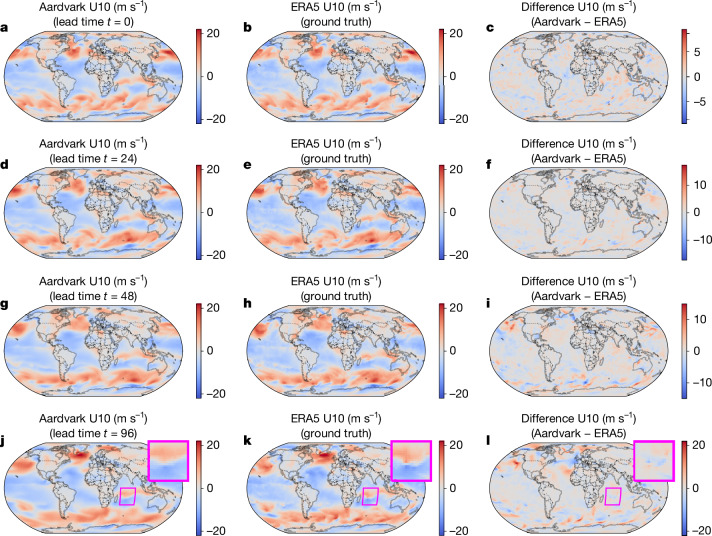
Example of Aardvark’s gridded forecasts for the U10 wind component. **a**–**l**, Plots of the initial condition (**a**–**c**) and subsequent forecasts (**d**–**l**) for U10, showing Aardvark’s prediction (**a**,**d**,**g**,**j**), the ERA5 ground truth^34^ (**b**,**e**,**h**,**k**) and the difference between the two (**c**,**f**,**i**,**l**). Lead time *t* = 0 corresponds to 00:00 on 11 January 2018. Aardvark correctly predicted the large-scale features for this variable and correctly predicted the formation and positioning of the tropical cyclone Berguitta (highlighted in the magent a boxes), which reached peak intensity on 15 January 2018 off the coast of Madagascar. We emphasize that the model made these predictions entirely from raw observations^40–48^, without any NWP products as input.

## Encoder module ablation

**Fig. 4:**
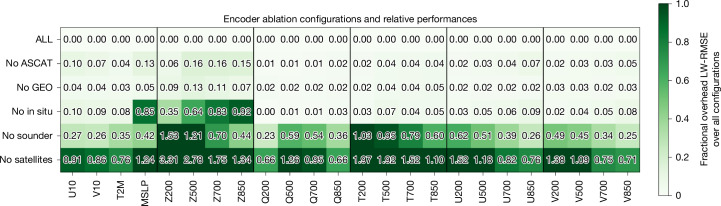
Encoder ablation experiments quantifying the impact of each data modality. The results of ablation experiments comparing the latitude-weighted RMSE (LW-RMSE) of the encoder trained with all data sources, both remote sensing^40–45^ and in situ sources^46–48^ (ALL) to other encoder configurations, including removing the scatterometer data (no ASCAT), removing the geostationary sounder data (no GEO), removing all in situ data (no in situ), removing all LEO sounder data (no sounder) or removing all satellite data (no satellite). We report the fraction of increase in LW-RMSE of each configuration relative to ALL.

A central innovation of the Aardvark Weather system is the estimation of an initial state from disparate data sources using the encoder module. With the volume and diversity of available observational modalities, two important questions arise. Which observational sources are most important for estimating each atmospheric variable, and how does each affect predictive performance? To investigate this, we conducted an ablation experiment to quantify the significance of each observational source in our encoder module. We removed different observational sources from the set of encoder inputs, retrained the encoder with this reduced set and evaluated it on the same test set as our original configuration, marked ‘ALL’ (Fig. 4). For example, the rows ‘no in situ’ and ‘no satellites’ correspond to removing in situ data and all satellite data, respectively, from the ‘ALL’ configuration. We report a fractional increase in the latitude-weighted RMSE relative to the ‘ALL’ configuration across all atmospheric variables for the initial condition generated at *t* *=* 0.

These results demonstrate that remote sensing data are of crucial importance in constraining the initial atmospheric state. Removing these data (no satellite in Fig. 4) and training with in situ observations lead to large skill reductions across all variables. Among different satellite modalities, low-Earth-orbit (LEO) sounder data are the most important. For example, removing these sounder modalities (no LEO) resulted in larger skill deterioration than, for example, removing scatterometer data (no Advanced Scatterometer (ASCAT)) or geostationary satellite data (no GEO). In situ observations are most important for surface variables. However, they also play a surprisingly large role in predicting geopotential, particularly at lower levels. These results indicate that for the future improvement of this system and the development of other end-to-end data-driven systems, LEO sounder data are the most important source to include, with in situ data providing an important complementary source to improve surface variables and geopotential forecasts. We provide full details of this experiment in Supplementary Information A.

## Evaluation of station forecasting

In the next stage of the weather prediction pipeline, global gridded forecasts are used as inputs to downstream models to produce a variety of products for end users. One such category of products is producing local forecasts. We focused on applying Aardvark Weather to predict 2-m atmospheric temperature and 10-m wind speed at off the grid station locations. Accurate local predictions of temperature are vital for the protection of public health during heatwaves and cold waves, in addition to agriculture and other use cases. Similarly, wind speed forecasts have a variety of end users, such as in wind energy, marine forecasting and fire weather forecasting. The modules for any desired downstream task can be substituted for this station forecasting module.

**Fig. 5:**
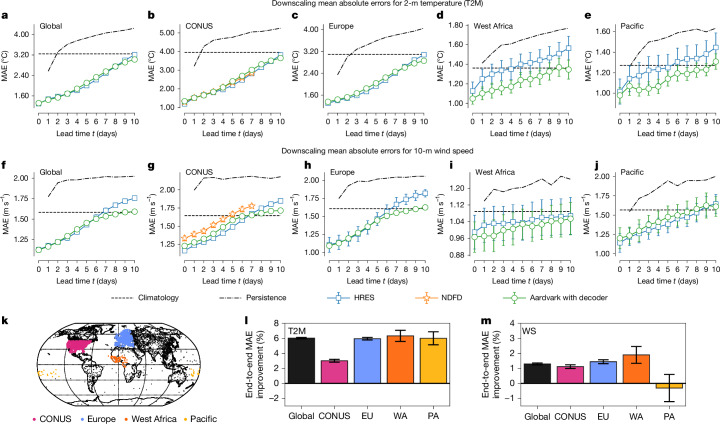
Station forecast performance and end-to-end fine-tuning improvements. **a**–**j**, The results of station forecasting for the held-out test set (2018) of HadISD data^46^, across different geographic regions (Global, CONUS, Europe, West Africa and Pacific) and predicted variables (2-metre temperature and 10-metre wind speed). Aardvark made predictions at spatial locations observed during training on temporally held-out data, but it can also generate predictions at any arbitrary station location. We compared Aardvark’s forecasts with two state-of-the-art NWP baselines: NDFD^36^ for CONUS. We also compared it against a version of HRES^33^ that we post-processed using a separate scale and bias term for each station. We report the mean performance of each system together with 98% confidence intervals in our estimate of the mean performance. **k**, Illustration of the definition of different geographic regions and the distribution of land stations we consider. **l**,**m**, Improvements from fine-tuning. We compared the predictions of Aardvark for lead time *t* = 1 day to those of its end-to-end fine-tuned counterpart for T2M and 10-m wind speed. We report the mean percentage of improvement in each variable by region (**k**) with 98% confidence intervals. ‘Global’ includes all stations (black and coloured). We emphasize that Aardvark produced its predictions entirely from raw remote sensing^40–45^ and in situ^46–48^ observations without any NWP products as input during the test time.

There are significant differences in how agencies in different countries produce forecasts for end users. In well-resourced countries, station forecasts are produced using global models followed by higher-resolution regional models out to a few days of lead time and statistical post-processing^36^. By contrast, in less well-resourced areas, although agencies have access to global products, they often do not have access to comparable infrastructure to run HRES, local NWP or post-process forecasts to a comparable degree^37^. With these considerations in mind, we report Aardvark’s performance across all stations globally but also break it down over four regions of particular interest: the contiguous United States (CONUS), Europe, West Africa and the Pacific (Fig. 5k). The USA and most European countries run both local NWP for shorter lead times, as well as sophisticated post-processing of both global and local products. By contrast, West Africa and the Pacific are regions in which many centres are less well equipped. Although some agencies in these regions run sophisticated NWP pipelines, others solely use raw HRES forecasts and issue operational forecasts for very short lead times^37^. We compared Aardvark against per-station persistence and climatology, as well as against two challenging baselines: station-corrected HRES and a full operational end-to-end baseline, the National Digital Forecast Database (NDFD) from the National Weather Service^36^. For a detailed description of the baselines, see Methods.

Figure 5 shows the mean absolute error (MAE) performance of Aardvark reported by variable and region. Globally, Aardvark generated skilful forecasts for both temperature and wind speed up to a lead time of 10 days, performing competitively with station-corrected HRES. For temperature, Aardvark was competitive with the station-corrected HRES over both CONUS and Europe. In addition, Aardvark matched the performance of the full operational NDFD baseline over CONUS. For lower-resource areas in West Africa and the Pacific, Aardvark outperformed the station-corrected HRES at all lead times. For 10-m wind speed, Aardvark had higher errors than the station-corrected HRES over CONUS and significantly outperformed the NDFD baseline. Over Europe, Aardvark had similar errors with the station-corrected HRES up to 4 days of lead time and outperformed it thereafter. Finally, Aardvark generally outperformed the station-corrected HRES over West Africa while performing slightly worse over the Pacific. In addition to these results, we compared Aardvark’s forecasts with a version of HRES that we post-processed using a separate scale and bias term for each station and NDFD for CONUS, demonstrating competitive performance on both variables (Supplementary Fig. 5).

## End-to-end tuning

End users of NWP products typically have a particular region and set of applications that are of interest. A powerful capability of Aardvark is its ability to tune the entire pipeline end to end to directly optimize for any desired quantity and region of interest. Optimizing the performance for a particular end-user product is challenging and expensive in a conventional NWP system. To explore this capability, we fine-tuned Aardvark to optimize predictions of 2-m temperature and 10-m wind speed at 1-day lead time globally and for each of the four regions. Although we focused only on these two variables, this is a powerful paradigm that can be applied anywhere there is uncertainty in the reanalysis training data, such as clouds and precipitation.

We observed that fine-tuning Aardvark yielded improvements both globally and in the specific regions of CONUS, Europe, West Africa and the Pacific (Fig. 5; bottom). For temperature, fine-tuning Aardvark resulted in large reductions in MAE of 6% over Europe, West Africa, the Pacific and globally, and an improvement of 3% over CONUS. For 10-m wind speed, small but statistically significant improvements of 1–2% were observed for all regions except the Pacific. To put these improvements into context, the last cycle update of IFS improved the surface variable scores in the range of 2–6% and took more than a year of development by a large team of scientists.

## Discussion

We have introduced Aardvark Weather, an end-to-end weather forecasting system, which is a data-driven system to tackle the entire NWP pipeline. Aardvark provides accurate forecasts that are orders of magnitude quicker to generate than existing systems without any reliance on NWP products at deployment time. Generating a full forecast from observational data takes approximately 1 s on four NVIDIA A100 GPUs compared to the approximately 1,000 node hours required by HRES to perform data assimilation and forecasting^38^ alone, before accounting for downstream local models and processing. In downstream tasks generating station forecasts of 2-m temperature and 10-m wind speed, Aardvark shows strong performance against operational NWP systems. Learning an end-to-end model offers the extra capability of optimizing the system to maximize performance over an arbitrary variable or region of interest, opening the door for the creation of inexpensive, individually tailored models for any region globally, in an automated and streamlined fashion.

End-to-end forecasting has significant potential for real-world effect. Compared with conventional NWP systems, machine learning systems are not only faster and computationally cheaper but are also significantly easier to improve and maintain. In conventional NWP, a new module, such as for a new parameterization or microphysics scheme, may take a team considerable time to build and integrate into the model. End-to-end data-driven systems, such as Aardvark, elegantly bypass this issue using a single model in place of this complex pipeline. The simplicity of this system makes it easier to deploy and maintain for users already running NWP and also opens the potential for wider access to running bespoke forecasts in areas of the developing world where agencies often lack the resources and expertise to run conventional systems. There is also significant potential in the demonstrated ability to fine-tune bespoke models to maximize predictive skill for specific regions and variables. This capability is of interest to many end users in areas as diverse as agriculture, renewable energy, insurance and finance.

To envisage how an end-to-end data-driven model such as Aardvark could be deployed operationally, it is necessary to consider the limitations of the current model and the concrete set of steps required to turn it into a fully fledged system. As with all current AIWP systems^1,6^, Aardvark does not yet run at the resolution of IFS. Further studies are required to increase the grid resolution and produce forecast ensembles through, for example, diffusion^2^. Other limitations centre around the use of observations. Further observational modalities will probably increase forecast skill. It is also important to consider how data from new instruments for which there are no training data available can be usefully integrated into the system. This can be accomplished by, for example, training on simulated data^39^. A further consideration is dealing with observation drift and other changes in data over time, which can be mitigated by regularly fine-tuning all modules with the most recent few months of data to adapt to changes in instrument characteristics.

The results presented in this study only scratched the surface of the potential of Aardvark Weather and end-to-end data-driven weather forecasting systems more broadly. Further capabilities can also be added by extending Aardvark to support several other forecast variables, both in its gridded forecasts and through its decoder module. For example, Aardvark can support a diverse range of decoder modules to provide different types of end user forecasts, such as hurricanes, floods, severe convection, fire weather and other extreme weather warnings. A further exciting avenue for future research is to use end-to-end systems at longer lead times to generate seasonal forecast products. More observational modalities would allow for the modelling of other components of the Earth system, such as atmospheric chemistry for air quality forecasts and ocean parameters for marine forecasts. We envision that Aardvark Weather will be a pioneer of a new generation of end-to-end weather forecasting systems to tackle these diverse tasks.

## Methods

### State estimation inputs

We selected several remote sensing and in situ observations as inputs to the atmospheric state estimation module. To ensure that no NWP system is required for the operational deployment of Aardvark, we selected only data that were available at either level 1B or 1C processing level^50^. Level 1B satellite data are calibrated and geolocated data, which means that the raw sensor measurements have been processed to correct for sensor and instrument biases but are still in the form of physical measurements, whereas level 1C satellite data are further processed to include radiometric and geometric corrections, making them ready for analysis with accurate geolocation and radiance values^50^. Other requirements for the inclusion of datasets are that they are available from 2007 to 2020 and in near real time to facilitate anticipated operational deployment. Where available for remote sensing products, we used fundamental climate data records, in which data from earlier generation sensors were homogenized to match the characteristics of current sensors, creating a consistent data record for training. Extended Data Table 1 provides a summary of all datasets that were used as inputs to the encoder module, including the type of instrument, orbit and platform (if applicable), as well as the data provider and data selection window that we used. For satellite instruments in LEO, it was necessary to include a longer window of observations to attain full global coverage. By contrast, station observations for all locations were available at *t* = 0 h. Therefore, adding data would be useful but is not necessary to achieve global coverage. As the data record was relatively short and overfitting is a concern, we decided to limit the data to the shortest window possible while retaining global coverage.

In situ observations from land stations, marine platforms and radiosondes were included. In situ land station observations measuring surface temperature (8,719 stations), pressure (8,016 stations), wind (8,721 stations) and dew point temperature (8,617 stations) at six hourly intervals were taken from the HadISD dataset^51,52^, provided by the UK Met Office. Marine in situ observations were taken from the International Comprehensive Ocean-Atmosphere Data Set^53^ provided by the National Oceanic and Atmospheric Administration. This dataset consists of observations from ships and buoys globally, from which five variables were included, namely 2-m air temperature, 10-m northward and eastward winds, sea surface temperature and mean sea level pressure. As observations were not taken precisely on the hour, all observations from *t* = −1 h to *t* = 0 h were included in the input. Upper-atmosphere observations of humidity, wind, geopotential and temperature were obtained from the Integrated Global Radiosonde Archive^54^, provided by the National Centers for Environmental Information. This dataset consists of radiosonde observations from 1,375 sites globally. Each record contained observations at several levels, of which we selected observations at the surface and at 200-, 500-, 700- and 850-hPa pressure levels. All profiles retrieved within the past 6 h, from *t* = −6 h to *t* = 0 h, were included in the input.

Because in situ observations were limited in geographic coverage, remote sensing observations from scatterometers and microwave and infrared sounders were included. Input data from satellites were ingested in the form of level 1 granules, each containing a 6-min slice of observations or orbits. Although, in principle, the Aardvark Weather system can handle these data in their raw form, for simplicity, data were first transferred to a regular 1° grid by nearest-neighbour interpolation, in which the most recent observation is maintained in cases where several observations are available for the same grid point.

Several scatterometers are currently operational worldwide, of which we used the ASCAT^55^ instrument aboard Metop-A, -B and -C. Data for this instrument are provided by the European Organisation for the Exploitation of Meteorological Satellites. ASCAT provides a triplet of three measurements of backscatter (*σ*_0_) from which operational centres retrieve the wind speed and direction, using a geophysical model function that solves for the two unknowns as a function of the *σ*_0_ triplet together with satellite metadata^56^. By contrast to this approach, we opted to simply include the raw *σ*_0_ values together with the metadata as channels to the encoder module, eliminating the complexity of the retrieval process. As all Metop satellites are in LEO, with a revisit time of approximately 24 h, the input to the state estimation module comprises the latest ASCAT observations available within the grid box from any of the three platforms on a regular 1.50° longitude–latitude grid from *t* = −1 day to *t* = 0 days.

In operational NWP, temperature and humidity profiles in the upper atmosphere are retrieved using infrared and microwave sounder instruments^57^. For this purpose, we included the Advanced Microwave Sounding Units A and B, Microwave Humidity Sounder instruments for microwave observations and the High-Resolution Infrared Radiation Sounder (HIRS)/4 for infrared observations. Together, these instruments comprise the Advanced TIROS Operational Vertical Sounder system that is used operationally to retrieve temperature and moisture profiles^58^. Data for these instruments are provided by the National Centers for Environmental Information. Observations for Advanced Microwave Sounding Units A and B, Microwave Humidity Sounder and HIRS are taken from the National Oceanic and Atmospheric Administration 15–19, Aqua and Metop-A satellites. In operational NWP systems, both the retrieved profiles and raw radiances are assimilated. Similar to ASCAT, profiles of the target variable were retrieved using a geophysical model function, taking in the raw radiances and satellite metadata and solving for the desired observational profiles. Again, we opted to input the raw radiances together with the satellite metadata directly into the state estimation module without relying on higher-level retrievals. As for ASCAT, the dataset consisted of the latest observations from *t* = −1 day to *t* = 0 days, taken within a grid box of a regular 1.50° longitude–latitude grid.

We augmented the Advanced TIROS Operational Vertical Sounder observations with data from the Infrared Atmospheric Sounding Interferometer (IASI)^59^, a hyperspectral infrared sounder. Data for this instrument were provided by the National Centers for Environmental Information. IASI captured data at a much higher spectral resolution than HIRS/4, with a total of 8,461 channels across three bands. To limit the input data volume, we took the leading 15 principal components across these channels, a technique demonstrated to lead to limited performance degradation in operational NWP systems. Data from IASI were available from October 2007 as opposed to January 2007 for the rest of the training set.

Although platforms carrying scatterometers and passive microwave sounder instruments in LEO provide HRES observations, they have the disadvantage of a lower temporal resolution. By contrast, geostationary satellites provide a very high temporal resolution although with more limited instrumentation. As the available channels on geostationary satellites vary geographically and with time, we opted to use a composite product, the Gridded Satellite dataset^60^, which provides homogenized retrievals of infrared and vapour window channels over standard geostationary platforms. Data for this instrument were provided by the National Climatic Data Center. For this data source, we included the image taken at *t* = 0 h.

To account for diurnal, seasonal and longer-term variations in the data, we included temporal information as input to both the encoder and forecasting modules. These channels consisted of $\sin \left(\frac{2{\rm{\pi }}d}{366}\right),\cos \left(\frac{2{\rm{\pi }}d}{366}\right),\sin \left(\frac{2{\rm{\pi }}h}{24}\right)$ and $\cos \left(\frac{2{\rm{\pi }}h}{24}\right)$, where *d* is the day of the year and *h* is the hour of the day. The absolute year was also included to account for any changes in data characteristics over the training record. To account for the effects of orography on the weather system, we included several sources of orographic information taken from the ERA5 dataset^34^ as static fields. The data were provided by the ECMWF. These were the geopotential at surface level, angle of sub-grid-scale orography, anisotropy of sub-grid-scale orography, slope of sub-grid-scale orography and standard deviation of orography.

### Pretraining

The modular structure of Aardvark leveraged ERA5 reanalysis data during the training phase to increase the length of the available data record. ERA5, or the fifth generation of the ECMWF reanalysis^34^, is a state-of-the-art global atmospheric reanalysis dataset. It provides comprehensive information on various meteorological parameters, such as temperature, humidity, wind and geopotential, covering the period from 1940 to the present. These data are provided by the ECMWF. From this, we elected to train on data from 1979 onwards, coinciding with the beginning of widely available remote sensing observations, which significantly improve the quality of the atmospheric reanalysis product.

### Baselines

For the global gridded forecast experiments, we compared the performance of Aardvark with four baselines: persistence, climatology, HRES and GFS. Persistence and climatology provide simple baselines for assessing whether a forecasting system is skilful. In persistence forecasting, it was assumed that the weather remains unchanged from *t* = 0 at all future lead times. For the climatology baseline, we used the climatology product from WeatherBench 2 (ref. ^33^). The predicted state was obtained by taking the mean value of all ERA5 observations from 1990 to 2017 for a given day of the year and hour using a sliding window length of 61 days.

The IFS and GFS are the two most widely used global operational NWP systems. As the focus of this study was on deterministic forecasting, we chose to compare our results with the HRES and GFS, deterministic runs at resolutions of 0.10° and 0.25°, respectively. These constitute challenging baselines for comparison with Aardvark Weather, which operates at a 1.50° resolution with just five vertical levels. For comparison with Aardvark, the HRES and GFS outputs were conservatively re-gridded to 1.50° resolution. In particular, we used HRES forecast data and ERA5 target data as provided by WeatherBench 2 (ref. ^33^), in which both datasets were coarsened to 1.50° resolution using first-order conservative re-gridding^61^. This procedure reduces the effects of aliasing, ensuring that Aardvark does not get an unfair competitive advantage because of distortions in the power spectrum that would occur from naive subsampling. To ensure that the GFS forecasts are compared fairly against Aardvark and HRES, we also applied conservative re-gridding to GFS. See Supplementary Information for further details on aliasing and its effects on signal spectra.

We considered four baselines for station forecasts. Persistence and climatology were calculated on the basis of station observations. For 2-m temperature, we calculated the daily climatology and for 10-m wind speed monthly. We further considered two more challenging baselines: station-corrected HRES and NDFD over the CONUS. As HRES is a gridded product, sub-grid-scale processes were not resolved. Therefore, we learned a bias correction individually for each station in the 2007–2017 training set and used this to correct the station forecasts on the 2018 test set. NDFD is produced by the National Weather Service in the USA and is a state-of-the-art local forecasting system^62^. Forecasts in the NDFD are created from an ensemble of more than 30 models^63^, including the IFS and GFS, together with HRES regional models at shorter lead times. The data from these systems are shown to human forecasters at different National Weather Service offices that create the final forecast. Our station forecasts were taken as the nearest-grid-box forecast from the final NDFD forecast, which was at approximately 2-km resolution. Therefore, NDFD constitutes an extremely challenging baseline, capturing the full complexity of operational forecasting pipeline.

### Evaluation metrics

For the global gridded forecasting experiments, we compared models on LW-RMSE. Given arrays of gridded target forecasts *y* and gridded target predictions $\hat{y}$, the LW-RMSE of variable $v$ is calculated as

1 $$\text{LW-RMSE}(\,y,\hat{y},v)=\frac{1}{B}\mathop{\sum }\limits_{b=1}^{B}\sqrt{\frac{1}{HW}{\sum }_{h=1}^{H}{\sum }_{w=1}^{W}{\alpha }_{h}{({y}_{bhwv}-{\hat{y}}_{bhvw})}^{2}}$$

where *b* indexes over *B* batch elements, $v$ indexes over *V* atmospheric variables, *h* and *w* are the index latitude and longitude coordinates over a grid with *H* points latitude-wise and *W* points longitude-wise, and ${\alpha }_{h}$ are the latitude weights, defined as

2 $${\alpha }_{h}=\frac{\cos {\theta }_{h}}{\frac{1}{H}{\sum }_{h=1}^{H}\cos {\theta }_{h}}$$

where ${\theta }_{h}$ is the latitude along the latitude-wise index *h*, so that their average is equal to 1. In machine learning, a (mini-)batch refers to a subset of the training dataset, typically used to compute a stochastic estimate of a model’s parameter gradients when performing gradient-based optimization. For the station forecasting experiments, we compared methods on MAE. Given arrays of station target temperatures *y* and predictions $\hat{y}$, MAE is calculated as

3 $${\rm{MAE}}(\,y,\hat{y})=\frac{1}{{BN}}\mathop{\sum }\limits_{b=1}^{B}\mathop{\sum }\limits_{n=1}^{N}|{{y}}_{{bn}}-{\hat{y}}_{{bn}}|$$

where $b$ indexes batch elements and *n* indexes the *N* stations in the forecast.

### Training objectives

Separate training objectives were used for each of the three modules. For all three modules, we normalized the targets by calculating the mean and standard deviation for each variable and level, aggregating across all grid points. In the encoder and processor modules, which involve several target variables, this normalization had an effect of implicitly weighting the variables, owing to the scaling applied during normalization. For the encoder module, we determined an extra weighting by first training the model using an LW-RMSE objective of the form

4 $$\text{SUM-LW-RMSE}(\,y,\hat{y})=\frac{1}{V}\mathop{\sum }\limits_{v=1}^{V}\text{LW-RMSE}(\,y,\hat{y},v)$$

Therefore, in the initial run, all variables were weighted equally. Next, weights ${\beta }_{v}$ were produced for each variable by taking the reciprocal of the LW-RMSE for each variable multiplied by a factor of 3 to generate weights within the range of approximately 0 to 1. The training objective for the encoder used these weights, giving the variable and LW-RMSE

5 $$\text{VLW-RMSE}(\,y,\hat{y})=\frac{1}{V}\mathop{\sum }\limits_{v=1}^{V}{\beta }_{v}\text{LW-RMSE}(\,y,\hat{y},v)$$

For the processor module, the training objective was SUM-LW-RMSE (equation (4)). However, the processor module was trained to predict residuals (see ‘Processor module’ below). We found that the implicit weighting that was applied through normalization worked well, and we did not further weight the variables individually. Finally, for the decoder module, the training objective was the same as for evaluation, that is, equation (3).

### Model architecture

Aardvark Weather is a neural process model^64^. Neural processes are a family of deep learning models that provide a flexible framework capable of learning with off the grid data, as well as missing and sparse data, and providing probabilistic predictions at arbitrary locations at test time. These characteristics are ideally suited to working with complex environmental data, such as in climate downscaling and sensor placement^65–69^.

Our specific architecture is a new member of the neural process family combining SetConv layers developed for the convolutional conditional neural process^24^, which handles off the grid and sparse data modalities and produces off the grid predictions, together with a vision transformer backbone that is currently used in state-of-the-art AIWP forecasting systems^70^. This provides scalability not currently attainable with standard transformer neural process models with attention-based encoders^71^ while still retaining the flexibility to handle diverse data modalities. Here we give details on the architectures of these modules, how they are trained and fine-tuned and how they are deployed. In the discussion that follows, note that the encoder, processor and decoder modules all receive auxiliary channels, such as temporal embeddings and orographic information, as input. For simplicity, we suppressed these channels in our exposition, but it should be understood that all three modules received them as inputs. We provide a complete list of all inputs and outputs to our models in Extended Data Table 2.

### Encoder module

The encoder module *E* takes raw observations as input, and outputs a gridded estimate of the initial state of each variable for the processor module. Let ${o}_{\tau }=\{{o}_{\tau ,1},\ldots ,{o}_{\tau ,N}\}$ be the set of observations corresponding to time *τ*, where each ${o}_{\tau ,n},$ corresponds to the observations and the corresponding metadata (such as viewing angle, solar elevation angle and observation time) of a single data modality. Each ${o}_{\tau ,n}=({x}_{\tau ,n},\,{y}_{\tau ,n})$ consists of a set of observations ${y}_{\tau ,n}$ and their corresponding longitude and latitude coordinates ${x}_{\tau ,n}$. Each data modality is either on the grid or off the grid and has a corresponding function ${\psi }_{n}$ to transform ${o}_{\tau ,n}$ into a gridded representation of fixed dimensionality. For gridded observations, ${\psi }_{n}$ consists of the addition of a masking channel to distinguish the missing data from the observed data in the grid. For off the grid observations, each ${\psi }_{n}$ consists of a SetConv layer^24^ with a learnable length scale. The SetConv layer produces a gridded representation of the data, as well as an accompanying density channel that carries information about the presence or absence of data, to handle irregularly sampled observations. The regular gridded representations of the modalities are concatenated to give a single gridded representation of dimension *C* × *H* × *W*, where *C* is the number of resulting channels, *H* is the number of latitude points and *W* is the number of longitude points. This representation of the input data is fed into the backbone of the module, consisting of a vision transformer ${V}_{{\rm{e}}}$ with a patch size of three, eight transformer blocks and a latent dimension of 512. Embeddings for each patch use a multi-layer perceptron following a previous study^27^. The encoder outputs the initial state estimate ${\hat{s}}_{\tau ,0}$ at time *τ* with dimensions of 24 × *W* × *H*, where 24 is the number of variables modelled in the forecasting module. Putting this together, we have

6 $${\widehat{s}}_{\tau ,0}=E({o}_{\tau })={V}_{{\rm{e}}}({\odot }_{n=1}^{N}{\psi }_{n}({o}_{\tau ,n}))$$

where ${\hat{s}}_{\tau ,0}$ is the estimated initial state corresponding to time *τ*, and ⊙ denotes concatenation. The encoder module is trained to predict ERA5 reanalysis targets using the VLW-RMSE (equation (5)) as its loss function. We trained the module for 150 epochs using AdamW with early stopping and a cosine learning rate scheduler starting at an initial learning rate of 5 × 10^−4^ and decaying to zero at the final epoch.

### Processor module

The processor module *P* takes the initial state estimate ${\hat{s}}_{\tau ,0}$ as input and outputs forecasts for lead times of 1–10 days. This module consists of ten separate vision transformers, ${V}_{{\rm{p}}}^{(1)},\ldots ,{V}_{{\rm{p}}}^{(10)}$, which were composed to produce gridded global forecasts at each of the ten lead times we considered. Here each ${V}_{{\rm{p}}}^{(i)}$ was designed to provide a 1-day forecast conditioned on the forecast of ${V}_{{\rm{p}}}^{(i-1)}$. This 24-h time step is a common configuration in AIWP models^71,72^ and was used here to avoid inconsistencies in assimilation procedures at the 06:00 and 18:00 UTC runs of IFS, which may disadvantage this baseline in the comparison^2^, and for computational tractability. All vision transformers have a patch size 5, latent dimension of 512 and 16 transformer blocks. To improve the modelling of interactions between variables, we added cross-attention between variables at the start of the network, as suggested in a previous study^73^. The processor is trained using a pretraining phase followed by a fine-tuning phase. Let ${\hat{s}}_{\tau ,{t}}$ be the ERA5 state corresponding to time *t* and lead time *τ*. During pretraining, the first vision transformer, ${V}_{{\rm{p}}}^{(1)}$, is trained to ingest ${s}_{\tau ,0}$ as input and predict the residual ${s}_{\tau ,1}\,-\,{s}_{\tau ,0}$ using the SUM-LW-RMSE loss (equation (4)). We pretrained ${V}_{{\rm{p}}}^{(1)}$ for 100 epochs using AdamW with a cosine learning rate scheduler starting at an initial learning rate of 5 × 10^−4^ and decaying to zero at 100 epochs. During the fine-tuning phase, we trained each vision transformer ${V}_{{\rm{p}}}^{(i)}$ to work with the estimated state produced by the previous transformer ${V}_{{\rm{p}}}^{(i-1)}$ as follows. Recall that ${\hat{s}}_{\tau ,0}$ is the estimated state produced by the encoder module. We started by training ${V}^{(1)}$ to predict ${s}_{\tau ,1}\,-\,{\hat{s}}_{\tau ,0}$ using the initial state ${\hat{s}}_{\tau ,0}$ as input. Once ${V}_{{\rm{p}}}^{(1)}$ has been fine-tuned, we computed ${\hat{s}}_{\tau ,1}={\hat{s}}_{\tau ,0}\,+\,{V}^{(1)}({\hat{s}}_{\tau ,0})$ and initialized the network ${V}_{{\rm{p}}}^{(2)}$ using the weights of ${V}_{{\rm{p}}}^{(1)}$. We then fine-tuned ${V}_{{\rm{p}}}^{(2)}$ to predict ${s}_{\tau ,2}\,-\,{\hat{s}}_{\tau ,1}$ using ${\hat{s}}_{\tau ,1}$ the previously estimated initial state as input. We proceeded sequentially in this fashion until all networks have been initialized and fine-tuned. This procedure can be regarded as an instance of the pushforward trick^74^. At deployment time, we composed the transformers to obtain a forecast for the desired lead time, that is

7 $${s}_{\tau ,t}=P({s}_{\tau ,0},t)={\widetilde{V}}_{{\rm{p}}}^{(t)}\circ \ldots \circ {\widetilde{V}}_{{\rm{p}}}^{(1)}({s}_{\tau ,0})$$

where ${\widetilde{V}}_{{\rm{p}}}^{(t)}(\cdot )=\,\cdot \,+{V}_{{\rm{p}}}^{(t)}(\cdot )$, and ${s}_{\tau ,0}=E({o}_{\tau })$ is the initial state produced by the encoder.

### Decoder module

The final step in the forecasting pipeline is the decoder module. For each lead time *t*, we trained a lightweight convolutional station forecasting module ${D}_{t}$, which takes the gridded estimated state ${s}_{\tau ,t}$, the target’s longitude–latitude coordinates *x* and auxiliary orographic information as inputs and produces predictions for the corresponding station temperature measurements ${y}_{\tau ,t}$. Each ${D}_{t}$ consists of a U-Net architecture^75^, followed by a SetConv layer that maps on-grid predictions to predictions at arbitrary station locations, followed by a multi-layer perceptron which incorporates the auxiliary orographic information, to produce local forecasts ${\hat{y}}_{\tau ,{t}}$. The U-Net consists of four encoder blocks (which consist of two-dimensional convolutions, BatchNorm layers, ReLU activations and MaxPool operations), followed by four decoder blocks (which consist of transpose two-dimensional convolutions, BatchNorm layers, ReLU activations and MaxPool operations). The encoder and decoder blocks have skip connections and channel dimensions (16, 32, 64, 128, 64, 32, 16, 1). We trained each *D*_*t*_ for 10 epochs using AdamW, with a learning rate of 1 × 10^−3^ and RMSE loss (equation (3)). To produce local forecasts at coordinates *x*, we computed

8 $${\widehat{y}}_{\tau ,t}={D}_{t}({s}_{\tau ,t},x)$$

where ${s}_{\tau ,t}$ is the global forecast defined in equation (7).

### End-to-end deployment

At deployment time, no ERA5 input is required to run the system. To obtain global forecasts, we composed the encoder and processor together and computed

9 $${\widehat{s}}_{\tau ,t}={P}_{t}\circ E({o}_{\tau })$$

where ${P}_{t}(\cdot )=P(\cdot ,t)$. If we want to produce local station forecasts, we compose the encoder, processor and decoder modules and compute

10 $${\widehat{y}}_{\tau ,t}={D}_{t}({P}_{t}\circ E({o}_{\tau }),x)$$

### Station forecasting baselines

We compared Aardvark against per-station persistence and climatology, as well as against two challenging baselines. The first of these is a station-corrected version of HRES: for each station, we selected the nearest grid point from the HRES 0.25° forecast and learned an affine correction (a scale and a constant bias) on a per-station basis to correct for systematic biases, which is a common and highly effective downscaling method^76^. Further, region-specific downscaling refinements are possible, for example, using a local nested NWP. These could potentially further improve the performance of NWP systems, so the station-corrected HRES results we presented should not necessarily be interpreted as the state-of-the-art in downscaling performance, but rather as a strong and globally applicable baseline. Second, over CONUS, we also compared against a full operational end-to-end baseline, the NDFD from the National Weather Service. NDFD forecasts are an archive of data from the National Weather Service offices produced by combining the output of several global and regional forecasting models, post-processing these and incorporating input from human forecasters^36^.

### End-to-end fine-tuning

To perform end-to-end fine-tuning, we composed the encoder together with the lead time *t* = 1 day processor and decoder modules, producing local station forecasts for lead time *t* = 1 day given by

11 $${\hat{y}}_{\tau ,1}={D}_{1}({P}_{1}\,\circ \,E({o}_{\tau }),x)$$

This composition produces a single machine learning model with inputs that consist of all raw observational sources of the encoder module and outputs that consist of the predictions of the decoder module. We then fine-tuned this composite mode, that is, all three networks, jointly with either 2-m temperature or 10-m wind speed station observations ${y}_{t,1}$ as the only targets, using the RMSE loss. Specifically, the fine-tuning procedure consists of loading the pretrained weights of the encoder, processor and decoder modules and performing stochastic gradient descent on the parameters of the three modules *E*, *P*_1_ and *D* to minimize the RMSE loss between the station forecast ${\hat{y}}_{\tau ,1}$ and its corresponding target ${y}_{t,1}$. We used AdamW and optimized all the parameters of the modules for 25,000 gradient steps with a constant learning rate of 5 × 10^−5^ and early stopping, as described by the following procedure.

During training, we stored checkpoints of our models to perform region-based model selection during evaluation. Specifically, every 1,000 fine-tuning gradient steps, we stored a copy of the model weights at that point in training, commonly referred to as a checkpoint. We then used the checkpoints to perform model selection on the basis of performance on a held-out validation set. Specifically, we evaluated each of the model checkpoints generated during fine-tuning on the validation data on the data from each of the regions we considered, namely global, CONUS, Europe, West Africa and the Pacific. For each region, we then selected the best checkpoint, as measured by performance on the validation set for that region, and evaluated this on the test data corresponding to the given region.

### Model size and training costs

All model training in this study was performed on a single virtual machine with four NVIDIA A100 GPUs. The encoder module contains approximately 31 million parameters and requires 13 h to train. The processor module contains approximately 54 million parameters and requires 8 h to train on ERA5 and 3 h to fine-tune using the output of the encoder module as the input. Each of the 11 decoder modules contains approximately 2 million parameters and takes approximately 30 min to train. End-to-end fine-tuning of the encoder, processor and decoder modules takes 2 h. Therefore, the total time to train the model is approximately 100 GPU hours.

### Further details

Further details on several aspects of this study, including supplementary figures and further discussion, are available in the Supplementary Information and rely on supplementary references^77–82^.

## Online content

Any methods, additional references, Nature Portfolio reporting summaries, source data, extended data, supplementary information, acknowledgements, peer review information; details of author contributions and competing interests; and statements of data and code availability are available at 10.1038/s41586-025-08897-0.

## Supplementary information

Supplementary Information Supplementary Sections A–E, including Supplementary Figs. 1–29.

## Figures and Tables

**Extended Data Table 1:**
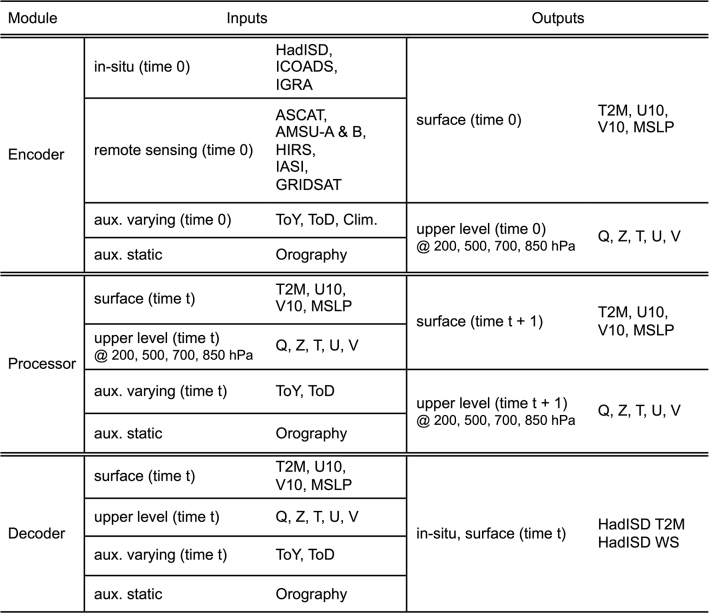
Listing of the inputs and outputs of each module

Raw data are passed to the encoder module which outputs predictions of the 24 prognostic variables on a global 1.50° grid at *t* = 0. This initial state is then input to the processor module to produce predictions for each of the prognostic variables at lead times of one to ten days on the same grid. Finally, the decoder module takes these global predictions to local predictions at station locations.

**Extended Data Table 2:**
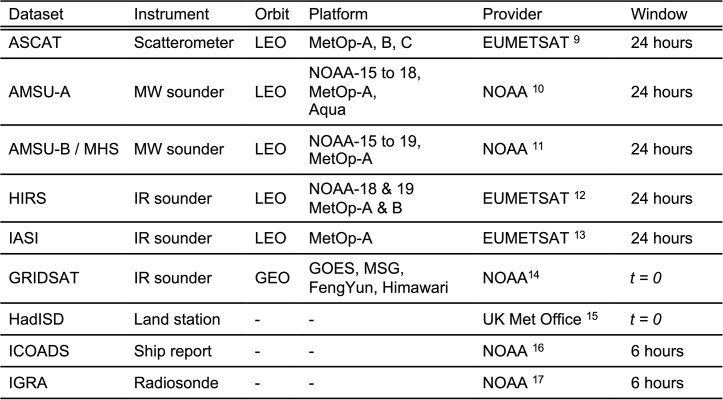
Summary of the observational datasets used to train Aardvark

Summary of the datasets, including the temporal window used in Aardvark. The acronyms “IR” and “MW” stand for infrared and microwave respectively.

## Data Availability

The dataset to run Aardvark Weather will be made available at https://huggingface.co/datasets/av555/aardvark-weather. All figures have been generated using a combination of the LaTeX TikZ package and the Matplotlib Python package^83^. All coastlines and borders drawn in the spatial plots in the main text (Figs. 1a, 3 and 5k) and Supplementary Information use the border and coastline functionality of the Matplotlib package.

## Code availability

The code used for training the models, the trained models, example test data and notebook examples for how to apply the models to make predictions will be made available on GitHub (https://github.com/annavaughan/aardvark-weather-public)^84^.
