# Supplementary material for: End-to-end data-driven weather prediction
Source: Nature. 2025 Mar 20;641(8065):1172–9. doi: 10.1038/s41586-025-08897-0 (PMC12119340; doi:10.1038/s41586-025-08897-0)
Supplement: Supplementary file 1 — Supplementary Sections A–E, including Supplementary Figs. 1–29. [file 41586_2025_8897_MOESM1_ESM.pdf]

nature portfolio

https://doi.org/10.1038/s41586-025-08897-0

Supplementary information

End-to-end data-driven weather prediction

In the format provided by the authors and unedited

Supplementary information

In this section we provide details of multiple important components of the Aardvark Weather system. Supplementary Information A details the design of the encoder module together with extended results of the ablation experiment. Full global forecast results are presented for latitude weighted RMSE in Supplementary Information B, with spectral plots, anomaly correlation coefficients and forecast activities in Supplementary Information C. Results of station predictions at held-out locations are presented in Supplementary Information D. Finally, Supplementary Information E provides additional state estimation and forecast plots for all variables.

A. End-to-end system design and observation ablations

As the design and implementation of the encoder is one of the most important components of the Aardvark Weather system we provide further details of the rationale behind our encoder design. Specifically, we discuss two major design choices. The first is our choice to tackle the state estimation stage with a "direct" neural network predictor, as opposed to the standard inferential approach that is used in traditional NWP. The second point we discuss is our decision to perform state-estimation solely from the data within the encoder's temporal window, instead of following the conventional approach that maintains a background atmospheric state and updates this with new data as these come in. Section A.2 presents the results of ablations of different data sources in the encoder, quantifying the impact that each of these has on model performance. We also discuss which possible observational modalities would be most promising for further improving end-to-end prediction systems.

Encoder design

We begin by discussing the distinction between conventional "inferential" approaches to state estimation used in traditional NWP, and contrast these with our "direct" approach that uses a neural network predictor, i.e. the encoder module.

Inferential approach. The standard approach to forecasting can be viewed as a probabilistic inference problem in a Markovian time-series model in which the prior predictive distribution (the forecast) and the likelihood (from the new observations) are combined to form the posterior over the latent atmospheric state in the assimilation step.

$$p(s_\tau | o_{1:\tau}) \propto p(s_\tau | o_{1:\tau-1}) p(o_\tau | s_\tau)$$

This inferential approach has several desirable properties. First, the assumptions being made about the variables and the approximations are explicit. Furthermore, it allows new observational sources to be included even when only scarce data are available. This approach also has important disadvantages. Making explicit modelling assumptions for all variables requires considerable expert knowledge and can be challenging to formulate. Moreover, approximations are required to perform the inference, for example retaining only the most likely atmospheric state. Finally, the framework does not easily allow the system to be optimised end-to-end.

Direct approach. In contrast to traditional data assimilation, the approach used in Aardvark directly optimises predictions of the initial atmospheric state from raw observations using a neural network. This network can learn complex relationships directly from data without requiring commitment to specific likelihood functions, including distributional assumptions such as Gaussianity. It is therefore relatively simple to add new data sources, provided that sufficient training data are available. A further advantage of

this approach is that it does not require approximate inference for which it is often hard to balance computational costs with acceptable accuracy. In addition, as we have demonstrated in this study, a direct approach based on parametric predictors opens the door to end-to-end training. This allows all parts of the pipeline to be directly optimised to the task of interest, thereby improving performance. More generally, neural network based predictors can also be pre-trained on a certain task, and then fine-tuned on a similar task of interest, a procedure which increases the effective dataset size and typically also improves performance[70]. The central disadvantage to this direct approach lies in whether sufficient training data are available to fit the model. Practically, this issue arises in scenarios where a new instrument is launched, or observational drift and other non-stationarities reduce the effective dataset size. Mature machine learning methods exist to handle these issues, using sim-to-real transfer [39, 77] to train on large simulated datasets before fine-tuning on scarce real data.

In choosing the direct approach, we take inspiration from broader work in machine learning demonstrating that with sufficient data availability end-to-end prediction outperforms statistical inference in complex models. For example, computer vision has moved away from treating vision-as-inverse-graphics, the inferential approach to vision, now relying on direct prediction methods [78]. Similarly, speech recognition has moved away from using probabilistic inference in a latent Markov model (Hidden Markov Models), instead relying on direct end-to-end approaches [79].

On the relationship between probabilistic inference and the direct forecasting approach

In this section we elaborate on the theoretical relationship between the conventional probabilistic inference approach and the direct forecasting approach. Aardvark Weather is founded on the framework of neural processes. Neural processes are a class of models that have been rigorously developed through a number of existing works[24, 64], which have demonstrated a number of desirable mathematical properties, including universal approximation guarantees[24], and have been demonstrated to outperform traditional approaches based on probabilistic inference, such as Gaussian processes, in data-rich settings. Interestingly, these papers also show that, once trained, neural processes implicitly perform optimal statistical inference internally, which includes weighting different information sources according to their uncertainty, a point that is related to ideas of meta-learning and amortised inference in the machine learning literature [80]. Specifically, it can be shown that, in the limit of large training data and large model size, Aardvark's prediction $\hat{s}_{\tau,t}$ for the atmospheric state at lead time t from initial time point τ is given by the true predictive mean

$$\hat{s}_{\tau,t} = \int \int s_{\tau+t} \, p(s_{\tau+t}|s_\tau) p(s_\tau|o_\tau) \, ds_{\tau+t} \, ds_\tau$$

where $p(s_\tau|o_\tau)$ is the true distribution over the initial atmospheric state s_τ given the observations o_τ and $p(s_{\tau+t}|o_\tau) = \int p(s_{\tau+t}|s_\tau) p(s_\tau|o_\tau) \, ds_\tau$ is the true forecasting distribution. In this way Aardvark learns to implicitly perform the two steps of classical data assimilation: state estimation $p(s_\tau|o_\tau)$ and forecasting $p(s_{\tau+t}|s_\tau)$. This argument suggests that rather than building in probabilistic inference directly using expert knowledge, this can emerge naturally by training to predict the atmospheric state.

Recurrent versus non-recurrent approaches to direct forecasting

Once committed to a direct parametric approach to state estimation, there are multiple potential architectures for the encoder. One natural idea would be to take inspiration from traditional DA systems and instantiate this module as a recurrent neural network (RNN). Unfortunately, RNNs present several challenges to train,

requiring a spin up phase and backpropagation through update cycles, and frequently suffering from difficulties with gradient instabilities. These are particularly challenging in the present case due to the size of the latent state. In contrast, a non-recurrent approach, i.e. one which does not rely on a recurrent state and instead uses observational data alone, is far simpler to train. This is especially relevant when the encoder module is composed with the processor and the downscaling modules in end-to-end training. The recurrent approach can be viewed as an instance of the non-recurrent approach that employs a memory and parameter efficient way to incorporate a very long time window of observations. In this light, the question over which architecture to opt for hinges on how long a history is needed to attain strong performance. Recent work indicates that using an observational window of observations of four days is sufficient [25], suggesting that non-recurrent approaches could feasibly be used to equal success as recurrent approaches, and rendering the challenges in training a recurrent system unnecessary.

Encoder ablation experiments

An important question to consider is the impact of each of the different observational modalities on the prediction of the initial gridded state. To make the comparison we retrain the encoder following the methodology described above dropping different subsets of variables and examine the resulting differences in performance. In total we compare six scenarios:

- All observations, corresponding to the encoder discussed in the main paper (ALL).
- All observations minus geostationary data (no GEO).
- All observations minus scatterometer data (no ASCAT).
- All observations minus in-situ observations (no in-situ).
- All observations minus LEO sounder data from ATOVS and IASI (no sounder).
- All observations minus all satellite data sources (no satellite).

The results of these experiments are shown in Figure 4. Excluding scatterometer data results in a reduction of around 10% in skill for U10 and V10, consistent with the known utility of this instrument for retrieving surface windspeed over the oceans. Small decreases in skill are seen across all variables. Excluding geostationary data results in a reduction of skill of 2-7% across all variables with the exception of upper level geopotential where skill reductions range from 7-13%. For in-situ observations, exclusion results in skill decrease across all variables with the exception of Q200 where the skill is unchanged. For other variables the decrease ranges from 1-10%, for all variables except MSLP and geopotential. For geopotential and MSLP the reduction in skill ranges from 35-92%, demonstrating the importance of in-situ observations in this setting. In contrast to the previous ablations where impacts are limited to a subset of variables, excluding the sounder data has a profound impact on all variables, with reductions in skill ranging from 21-153%. Impacts are particularly pronounced at upper levels where limited in-situ data are available to constrain predictions. In the final ablation, we exclude all satellite and train the encoder only on in-situ observations excluding all satellite data sources. This results in a substantial reduction in performance across all variables, with larger degradation of performance across all levels.

We now turn to interpreting what these results mean in the context of improving the encoder and extending end-to-end forecasting by including further observational sources. The degradation of the representation of MSLP and geopotential with the removal of in-situ obs indicates that further sources of data for this variable

could lead to significant improvements in performance. An ideal candidate for this would be the inclusion of Global Positioning System Radio Occultation (GPS-RO) data, which provide information on temperature, pressure, and humidity profiles. Inclusion of further data on upper level wind profiles would likely also enhance performance for U and V. This could be obtained through further in-situ data from aircraft observations, or alternatively through lidar instruments such as the Aeolus wind profiler.

B. Full global forecast results

Here we provide full results for the latitude weighted RMSE performance of Aardvark together with our baselines, namely climatology, persistence, GFS and HRES, across all variables predicted by Aardvark.

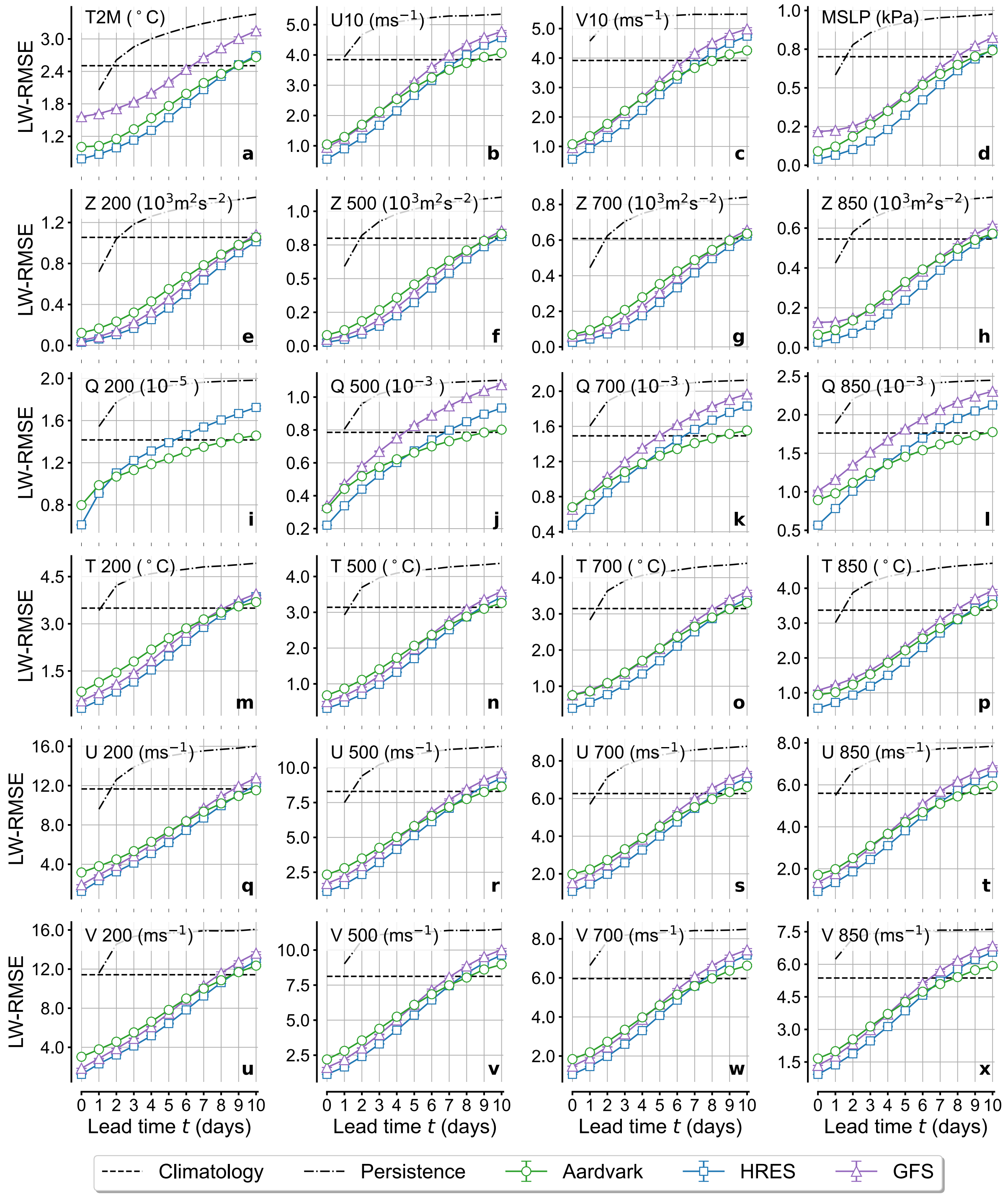

Figure 1. Reporting latitude-weighted RMSEs across all variables. *LW-RMSE performance of Aardvark, HRES [33] and GFS [49], using ERA5 [34] reanalysis data as the ground truth, on the held out test set (2018), for the surface variables (a-d) and the upper level variables (e-x), as a function of lead time t. The error bars show 98% confidence intervals in the estimated mean. Most error bars are too small to see in this plot. At lead time t = 0, Aardvark predicts the initial atmospheric state from observational data alone. The error at t = 0 is the error in the initial state. HRES has non-zero error at t = 0, as it is compared to ERA5 reanalysis ground truth.*

C. Spectral, correlation coefficients and activities

Here we give a complete set of spectral plots across all variables modelled by Aardvark, as well as the power spectra of ERA5 at 0.25°, as well as ERA5 and the HRES and GFS analyses ($t = 0$) at 1.50°, as provided by WeatherBench 2. We then give anomaly correlation coefficient (ACC) plots for all variables, comparing Aardvark and HRES. We also discuss observations around spectral smoothing.

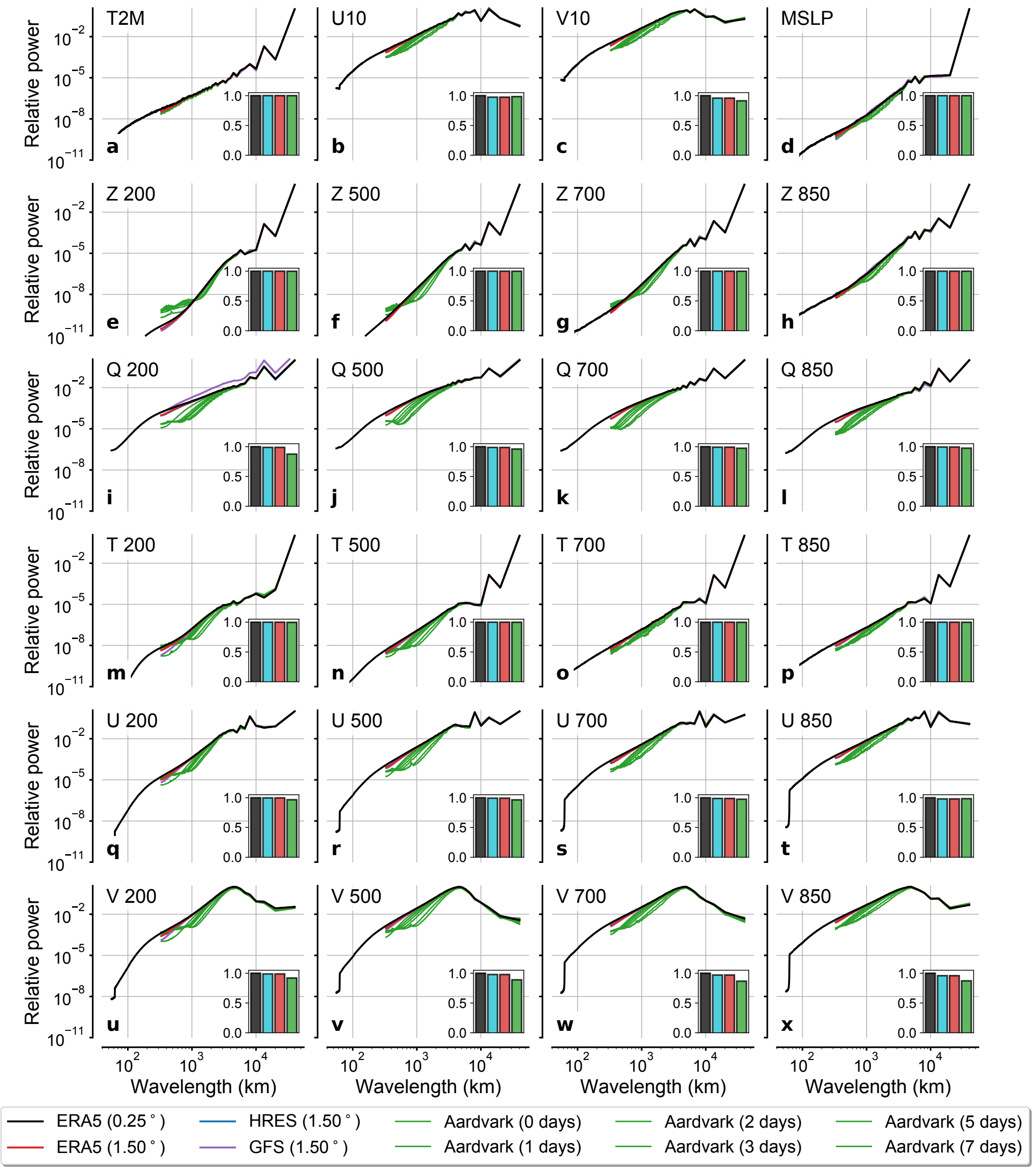

Figure 2. Spherical harmonic power spectra. *Spherical harmonic power spectra across all variables modelled by Aardvark (green), together with the corresponding spectra of the ERA5 [34] reanalysis at 0.25° (black) and 1.50° (red), the HRES [33] analysis at 1.50° (blue) and the GFS [49] analysis at 1.50° (purple). For ease of comparison, the spectrum of each variable has been rescaled so the maximum mode of ERA5 at 0.25° has unit power. We refer to these as relative powers. The ERA5 and HRES data at 1.50° have been generated by WeatherBench 2 by applying conservative re-gridding to higher resolution fields. The inset bar plots show the normalised total power content for the spectra of: ERA5 at 0.25° (black), ERA5 at 0.25° after removing the high frequency modes that are lost during coarsening, ERA5 at 1.50° after conservative regridding (red) and Aardvark's analysis state at t = 0. Conservative re-gridding drops a small fraction of power. Aardvark loses relatively small fractions of the spectrum's total power for most variables. The same holds for the spectra of ERA5 at 1.50° and at 0.25°, across most wavelengths.*

Power spectra and conservative re-gridding

The ERA5 and IFS data we use in this work are obtained from WeatherBench 2, which has used conservative re-gridding to compute its 1.50° products. Conservative re-gridding[61] minimizes the impact of aliasing when reducing the spatial resolution, because it uses mean-smoothing when coarsening, as well as re-gridding weights to account for overlapping grid cells. As a result, the power spectra of ERA5 at 0.25° (black) agree closely with those of the re-gridded product 1.50° (red) in Figure 2, modulo the modes that are entirely dropped in the coarser fields. In addition, again due to conservative re-gridding, the HRES and GFS analysis spectra (blue and purple) match that of ERA5 extremely closely, with the spectra often being indistinguishable.

To quantify the effect of dropped modes and spectral smoothing more precisely, we computed the total power lost from the spectrum of ERA5 at 0.25° due to dropping high-frequency modes (from coarsening to a lower resolution grid) and due to spectral smoothing (from applying overlapping cell weighting in conservative regridding). We report these results in the inset figures, which show the normalised total power for: ERA5 at 0.25° (black), ERA5 at 0.25° after removing the high frequency modes that are lost when coarsening to 1.50° (cyan), ERA5 at 1.50° after conservative re-gridding (red), and Aardvark at 1.50° (green). We observe that the overall power lost by conservative re-gridding, both due to dropped modes as well as spectral blurring, is negligible. We also observe that the total spectral power of Aardvark closely matches that of the reanalysis data across all variables. From the aforementioned results, we conclude that the NWP methods are not in fact being disadvantaged by aliasing, and that our comparison with Aardvark is a fair one.

Spectral smoothing and anomaly correlation coefficients

While Aardvark accurately captures the dominant modes of all variables, it also displays some degree of spectral smoothing, which increases with lead time. Spectral smoothing is a commonly occurring phenomenon in ML-based forecasting systems and it occurs for two main reasons. First, deep learning methods are known in general to suffer from spectral bias even when there is no predictive uncertainty due to the structure of the models and dynamics of stochastic gradient-based optimisation [81]. Second, when there is uncertainty in the predictions, models trained with a deterministic (R)MSE loss [1,6,70] will over-smooth. Smoothing occurs because the optimal prediction under an (R)MSE loss is the mean of the true predictive distribution of the forecasted variable (e.g. the atmospheric variable grid at time $t + \delta t$ given the conditioning variables (e.g. the grid at time t). Since the high-spatial frequency components of the meteorological variables are less predictable, in order to minimise the (R)MSE loss in the presence of uncertainty, the model hedges its bets and blurs its predictions, eventually converging to the climatological mean at the longest lead times. Employing a deep generative model, such as those based on diffusion[2], would enable samples to be generated from the predictive distribution which maintain realistic high-frequency content.

Anomaly correlation coefficients

Strictly speaking, the aforementioned blurring effect can make the forecasts of ML-based systems and NWP deterministic systems less comparable at longer lead times. This is because ML-based forecasting systems effectively target a different quantity, which more closely resembles an NWP ensemble mean. While smoothing can be addressed by generative forecasting models [2], deterministic forecasts are still skillful and arguably remain valuable despite blurring. To further assess the extent to which the predictions of Aardvark revert to a climatological mean, we evaluate its ACCs. For simplicity, consider a fixed forecast initialisation time and a fixed lead time, and let true targets and model predictions for variable v be y_{hwv} and $\hat{y}_{hwv}$ at the point with longitude index h and latitude index v. Let c be the value of the corresponding climatological

mean at the appropriate forecast time (i.e. at the forecast initialisation plus lead time). The latitude-weighted ACC of the atmospheric variable v is defined as

$$\mathrm{ACC}(y, \hat{y}, v) = \frac{\sum_{h=1}^{H} \sum_{w=1}^{W} \alpha_h (y_{hwv} - c_{hwv})(\hat{y}_{hwv} - c_{hwv})}{\sqrt{\sum_{h=1}^{H} \sum_{w=1}^{W} \alpha_h (y_{hwv} - c_{hwv})^2} \sqrt{\sum_{h=1}^{H} \sum_{w=1}^{W} \alpha_h (\hat{y}_{hwv} - c_{hwv})^2}} \quad (12)$$

In order to compute aggregated ACCs, for each variable and lead time, we compute the ACC for each individual forecast initialisation time in our test set and report the mean ACC together with its confidence intervals. The ACC ranges between 1 and -1, with an ACC of 1 denoting perfect correlation between the model anomaly and the reanalysis reference anomaly. An ACC of 0 indicates an uninformative forecast, which typically occurs because the forecast anomaly $y_{hwv} - c_{hwv}$ is close to zero, i.e. the model is predicting the base climatology. We also note that an ACC of 0 can also occur with a non-zero anomaly, provided it is fully uncorrelated to the base anomaly, however this scenario is not typically encountered, with reversion to climatology being the typical behaviour. Finally, a negative ACC indicates a misleading forecast, whereby the predicted anomaly is in direct disagreement with the reference anomaly. In SI Figure 3 we observe that while Aardvark typically demonstrates lower ACCs than HRES, it retains skillful predictions with respect to climatology and does not fully revert to the climatological average even up to lead times of even up to 10 days. This provides evidence that the competitive performance of Aardvark at moderate lead times is not due to a reversion to a climatological mean (in this case the ACC scores would rapidly decay to zero), but rather represents meaningful forecasting skill.

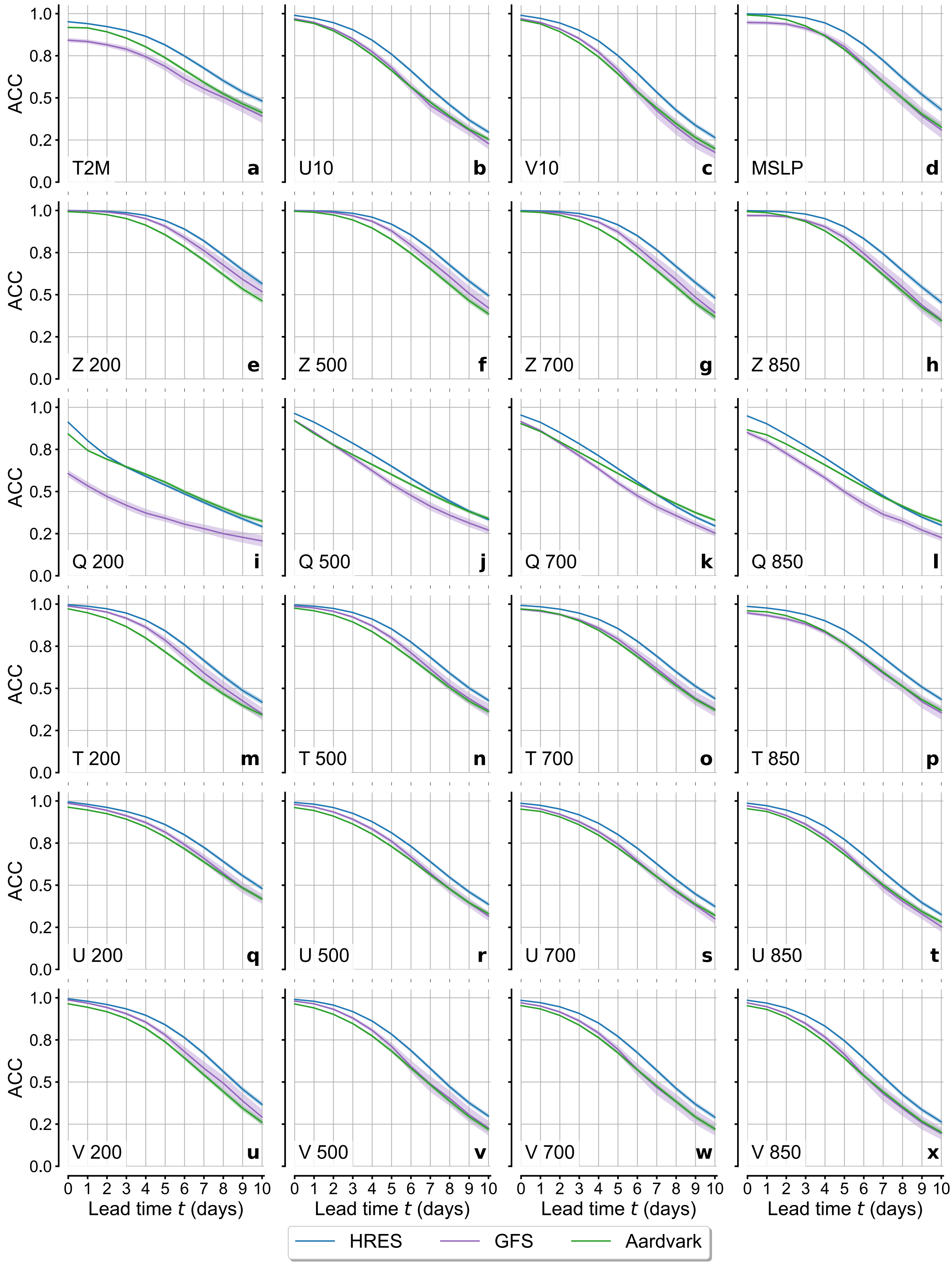

Figure 3. Reporting anomaly correlation coefficients across all variables. *Anomaly correlation coefficients (ACC) for the forecasts of Aardvark, compared against HRES [33] and GFS [49]. All model anomalies are computed using a base climatology field, and the reference anomaly is computed against ERA5 reanalysis [34], at a 1.50º resolution. We report the mean ACC for 2018, together with 98\% confidence intervals in the mean (which are very small in this plot).*

Forecast activities

Forecast smoothing is a common phenomenon in machine learning weather forecasting models that are trained, for example, with an RMSE loss[1,6,70]. As discussed above, smoothing typically occurs for two main reasons. The first is that the optimal prediction that minimizes the RMSE on average, is the mean of the predictive distribution of the variable being forecasted. At shorter lead times, there is little uncertainty in this distribution, but due to the chaotic nature of the weather system, uncertainty increases at longer lead times. In order to minimise the RMSE, ML models hedge their bets and predict smoothed-out forecasts that emulate the behaviour of the ideal ensemble mean. The second factor that introduces smoothing is that the RMSE loss affects the training dynamics of neural networks [81]. In particular, the most important frequency components for minimising the RMSE loss are those with the highest power content, which are the low-frequency components in the case of the atmosphere. Deterministic neural network training has been shown to focus on optimising these lower-frequency components at the expense of higher frequency components, thereby introducing smoothing [82].

While the spherical harmonic power spectra in Figure 2 help assess the extent of forecast smoothing, a metric which directly quantifies this effect is forecast activity. Forecast activity is defined as the latitude weighted RMSE between a forecast, and an appropriate climatological mean. For example, given a forecast array y indexed by height $h = 1, \dots, H$, width $w = 1, \dots, W$, and variables $v = 1, \dots, V$, and a corresponding climatological mean c, the forecast activity is defined as

$$\mathrm{ACT}(y, v) = \sqrt{\frac{1}{HW}\sum_{h=1}^{H}\sum_{w=1}^{W}\alpha_h(y_{hwv} - c_{hwv})^2} \tag{13}$$

To aggregate the forecast activities across initialisation dates for a fixed lead time, we compute the mean activity as well as the standard error in the estimate of the mean, and report the results in SI Figure 4. Note that we report the forecast activity for each variable and lead time relative to HRES, i.e. we normalise by the forecast activity of HRES. Therefore, a relative forecast activity of 1 indicates an activity higher than that of HRES, while forecast activities close to 0 mean that a forecast is close to a climatological average. While some reduction in forecast activities is visible in Aardvark's forecasts, the relative activity remains relatively high across most variables and lead times. The most significant reduction in forecast activity occurs in specific humidity (Q), where the relative forecast activity decays down to about 0.60-0.70. For all other variables, relative forecast activity remains around or above 0.80. These scores provide direct evidence that while some smoothing is taking place in Aardvark's forecasts, the forecasts maintain non-trivial information up to the longer lead times that we have considered.

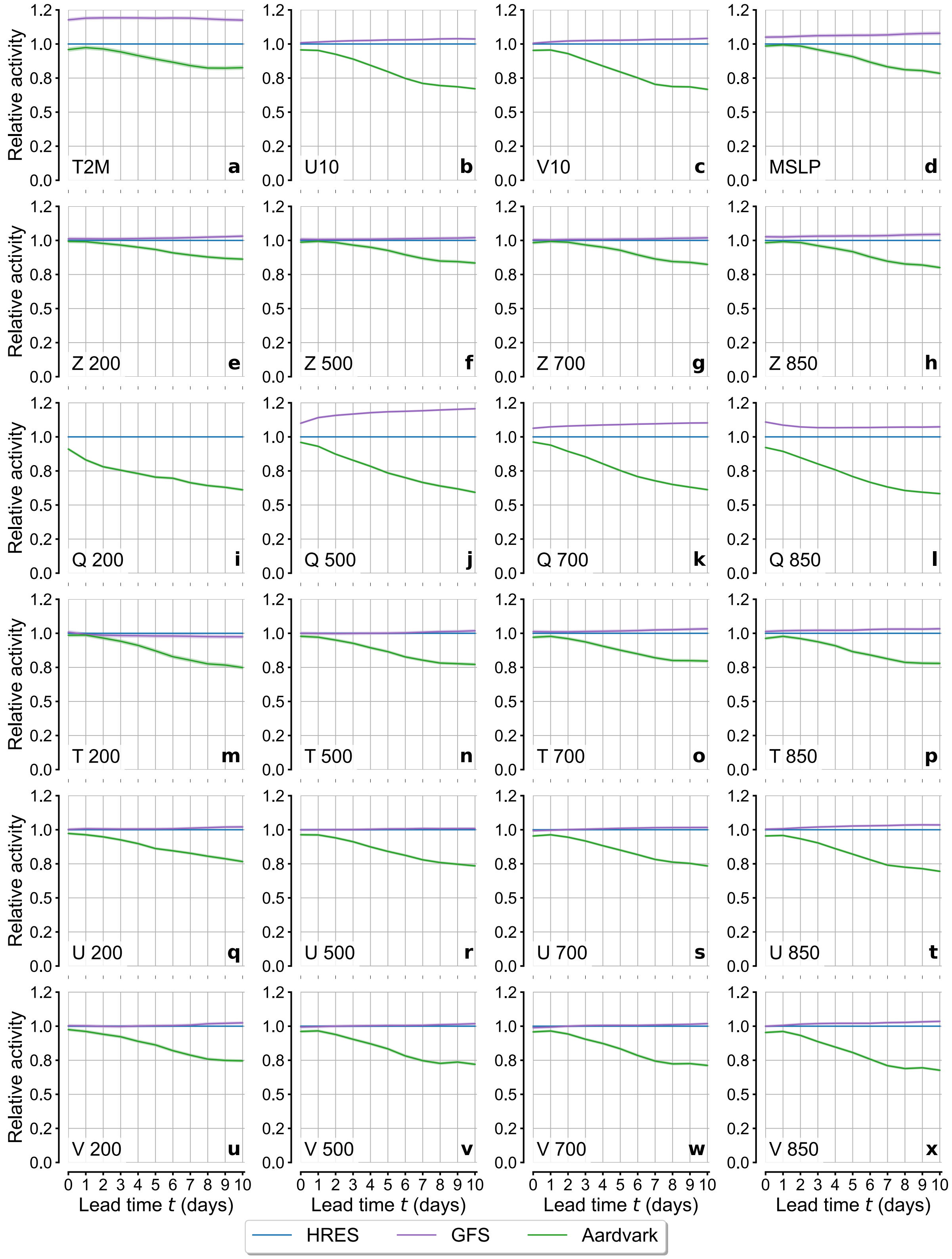

Figure 4. Reporting forecast activities across all variables. *Forecast activity of Aardvark, HRES [33], and GFS [49]. Forecast activity is defined as twice the LW-RMSE of the forecast against a reference climatology field, here the ERA5 climatology [34]. Activities are scaled and centered relative to the HRES activity. We report the mean activities for 2018, with 98% confidence intervals in the mean.*

D. Station prediction at spatially held out locations

One important point to consider when performing statistical downscaling is whether the stations at which downscaling is performed are observed at training time. This is because station observations can be influenced by features such as fine-scale orography, or other local factors. It is therefore possible that a model could learn to successfully generalise station forecasts *across time*, but fail to generalise *across space*. In other words, a model may be able to memorise statistical patterns for individual stations based on station location, but fail to produce accurate forecasts at new stations. While generalisation across time is a highly useful capability in its own right, it is also important to assess generalisation across space.

To assess the capability of Aardvark's encoder to generalise across space, we performed a held-out station evaluation. In this evaluation, we use a global, geographically diverse set of stations, which has been selected by dividing the globe in equal 1.00° by 1.00° latitude-longitude boxes, and selecting a maximum of two stations at random from each box. These stations are held out from the model during training, and are also held out from the encoder at deployment time, so they do not provide any information to the model.

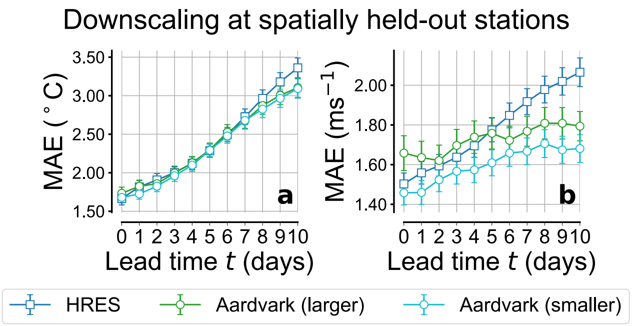

***Figure 5. Station forecast performance on held-out stations**. Performance of Aardvark on a global, diverse set of held out HadISD stations [46] for 2-metre temperature (T2M) and 10-metre wind speed (WS), against HRES [33]. We compare two variants of our downscaling module, with identical convolutional architectures but different numbers of convolution channels. Note that because the stations are held-out from the training set, the per-station climatology and persistence baselines are not defined, and per-station bias correction cannot be applied to HRES.*

We compare two variants of our encoder module. The first variant, referred to as "larger," is the module described in the "Decoder module" section. The second variant, referred to as "smaller," is architecturally identical to the first module, except all dimensions of the UNet encoder and decoder blocks have a factor of 8 fewer channels. The results of evaluating Aardvark on these stations are provided in SI Figure 5. We note that, since the station data are held out, the per-station climatology and persistence baselines are not defined. For the same reason, it is not possible to apply the same per-station scaling and bias correction which we applied to our HRES baseline, as described in section 9 "Performance comparison: station forecasting". We observe that Aardvark produces results competitive with those of HRES, for both 2-metre temperature as well as for 10-metre wind speed. We also note that using a decoder with fewer parameters does lead to a modest, but statistically significant performance improvement in terms of wind speed. Overall, we conclude that Aardvark is able to successfully generalise to unseen stations, with a performance that is as good, if not better, than that of HRES. That being said, it should be noted that this HRES baseline should not necessarily be interpreted as the state-of-the-art station forecasting method. Local NWP refinement schemes, such as nested simulations, tailored to specific geographic regions are likely stronger than HRES in predicting 2-metre temperature and 10-metre wind speed. We have nonetheless chosen to use HRES as an indication of NWP performance in this generalisation experiment, because we are using a global and diverse set of stations that is not covered by any single local NWP model.

E. Additional initial state estimation and forecast plots

Here we include additional plots of estimated states and forecasts produced by Aardvark. Lead time $t = 0$ corresponds to the estimated initial state. We set $t = 0$ to be the 11th of January 2018, corresponding to the formation of tropical cyclone Berguitta.

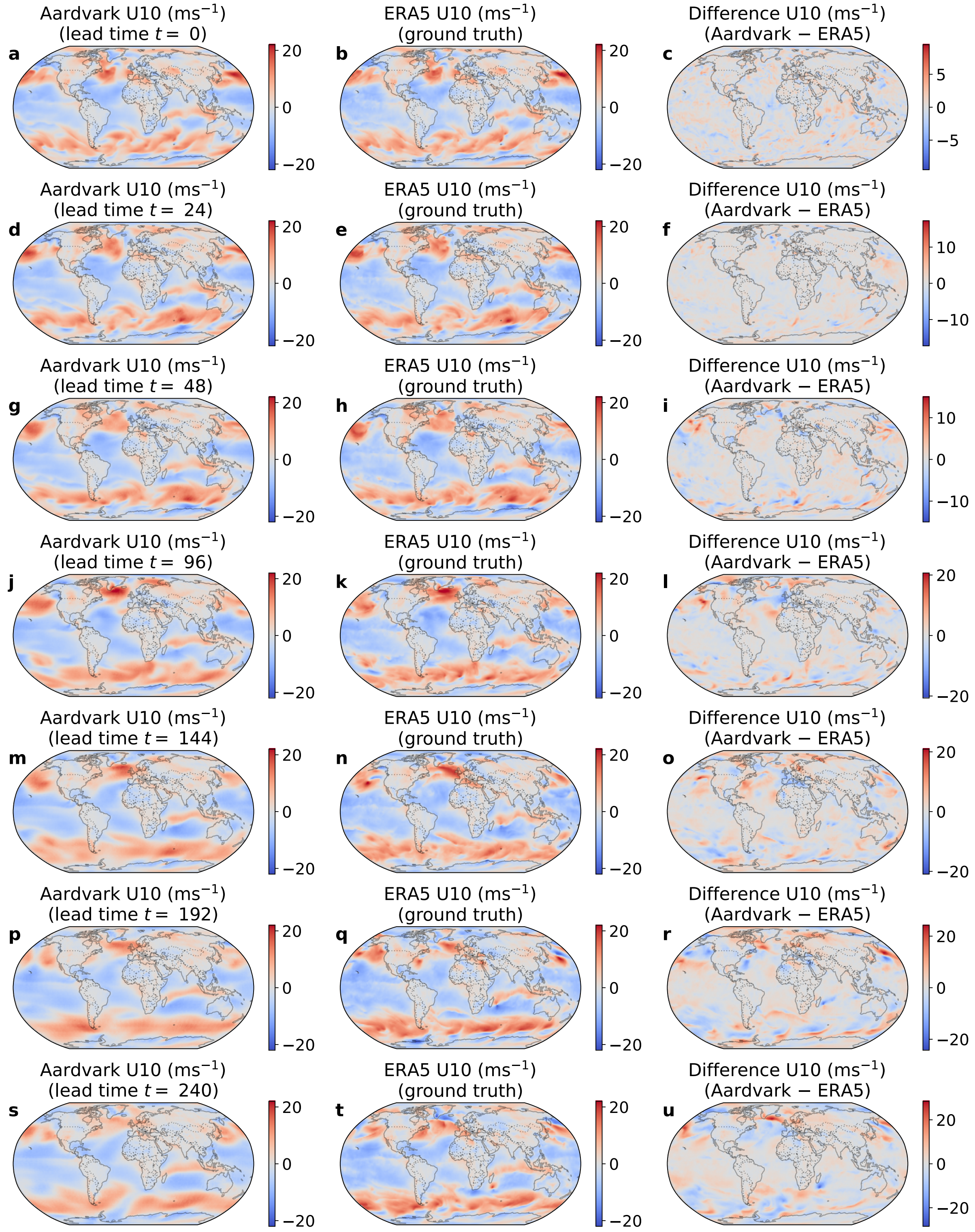

Figure 6. Illustration of Aardvark's forecasts against ERA5 [34]***.*** *Note t = 0 is 11th January 2018.*

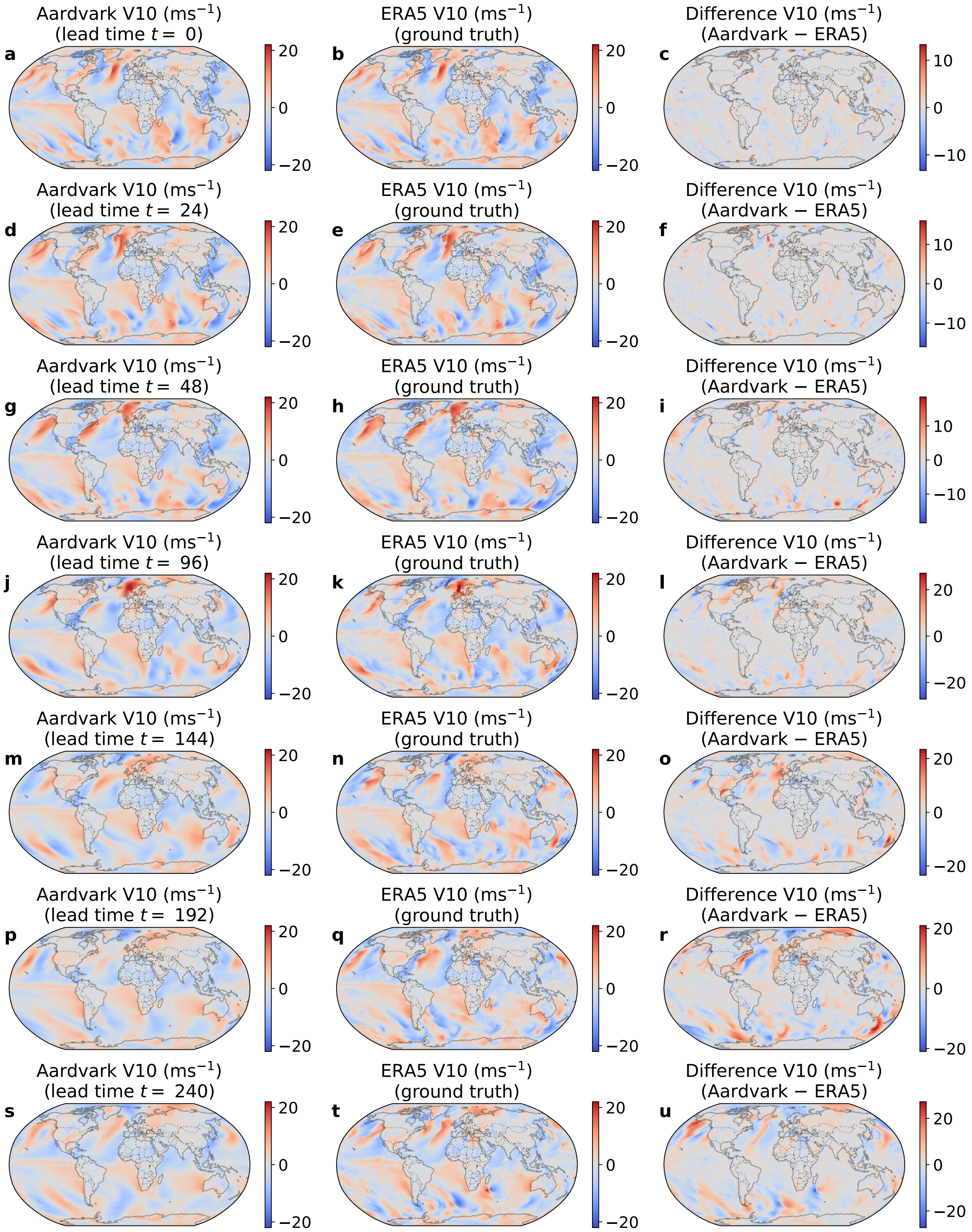

Figure 7. Illustration of Aardvark's forecasts against ERA5 [34]***.*** *Note t = 0 is 11th January 2018.*

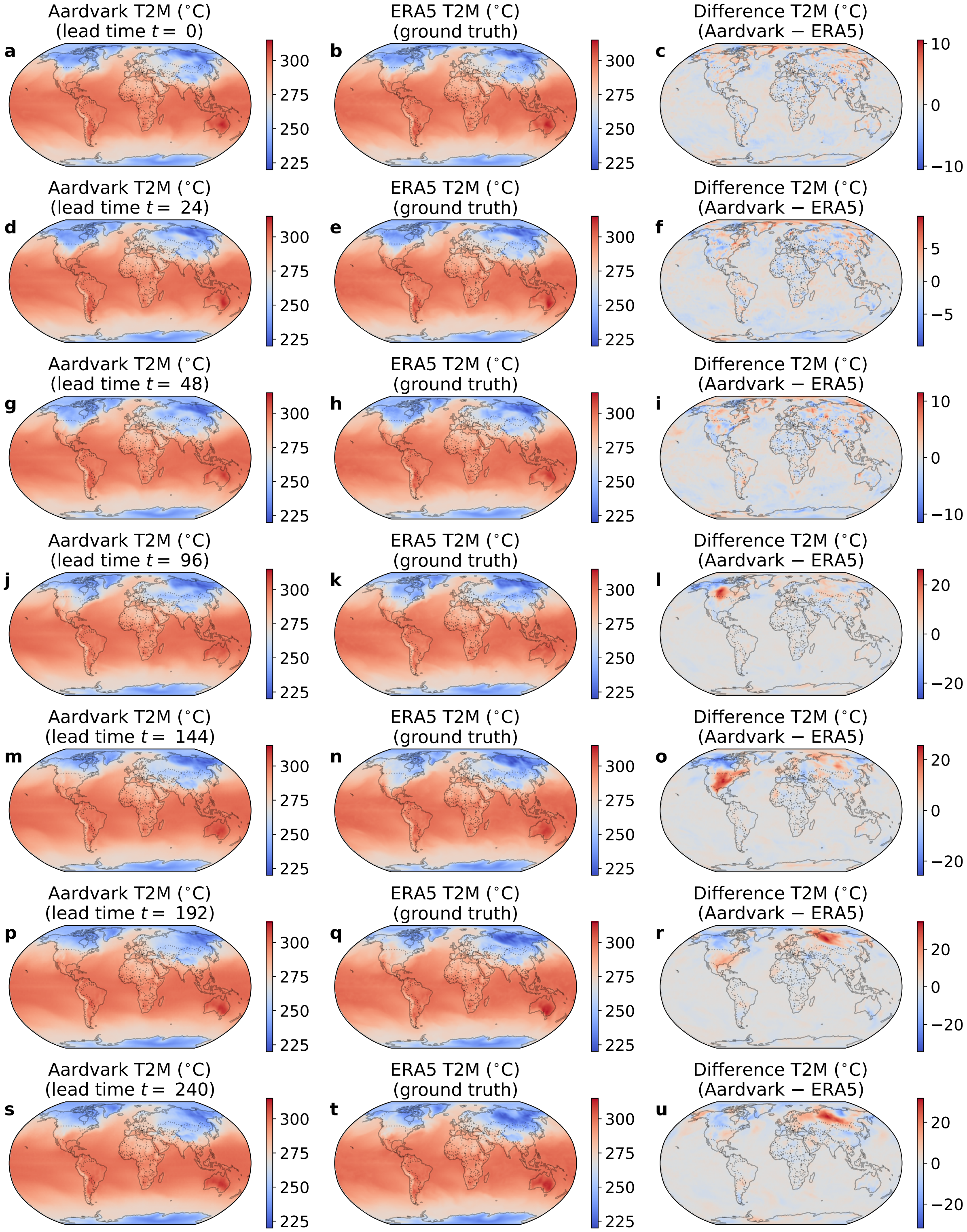

Figure 8. Illustration of Aardvark's forecasts against ERA5 [34]. *Note t = 0 is 11th January 2018.*

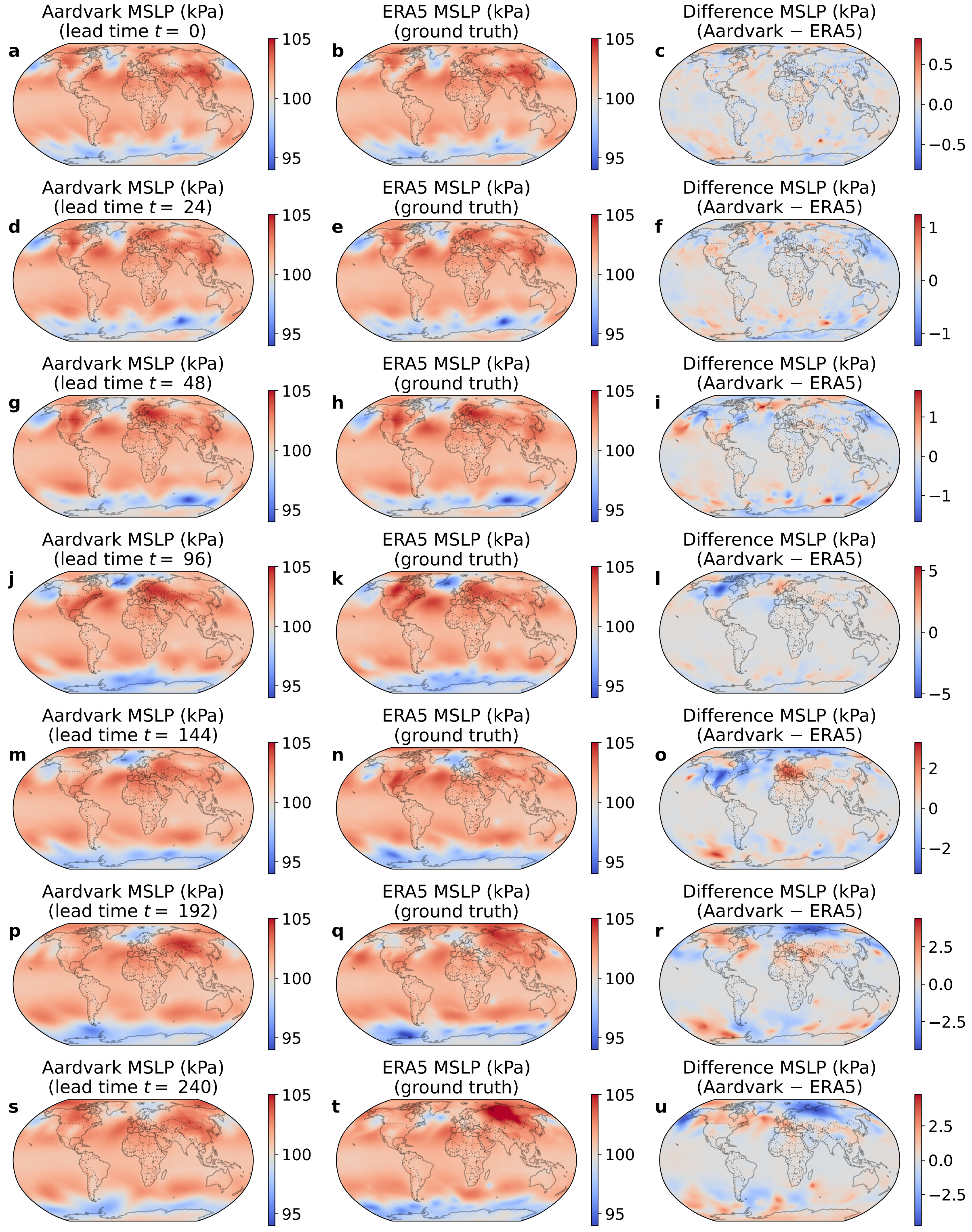

Figure 9. Illustration of Aardvark's forecasts against ERA5 [34]***.*** *Note t = 0 is 11th January 2018.*

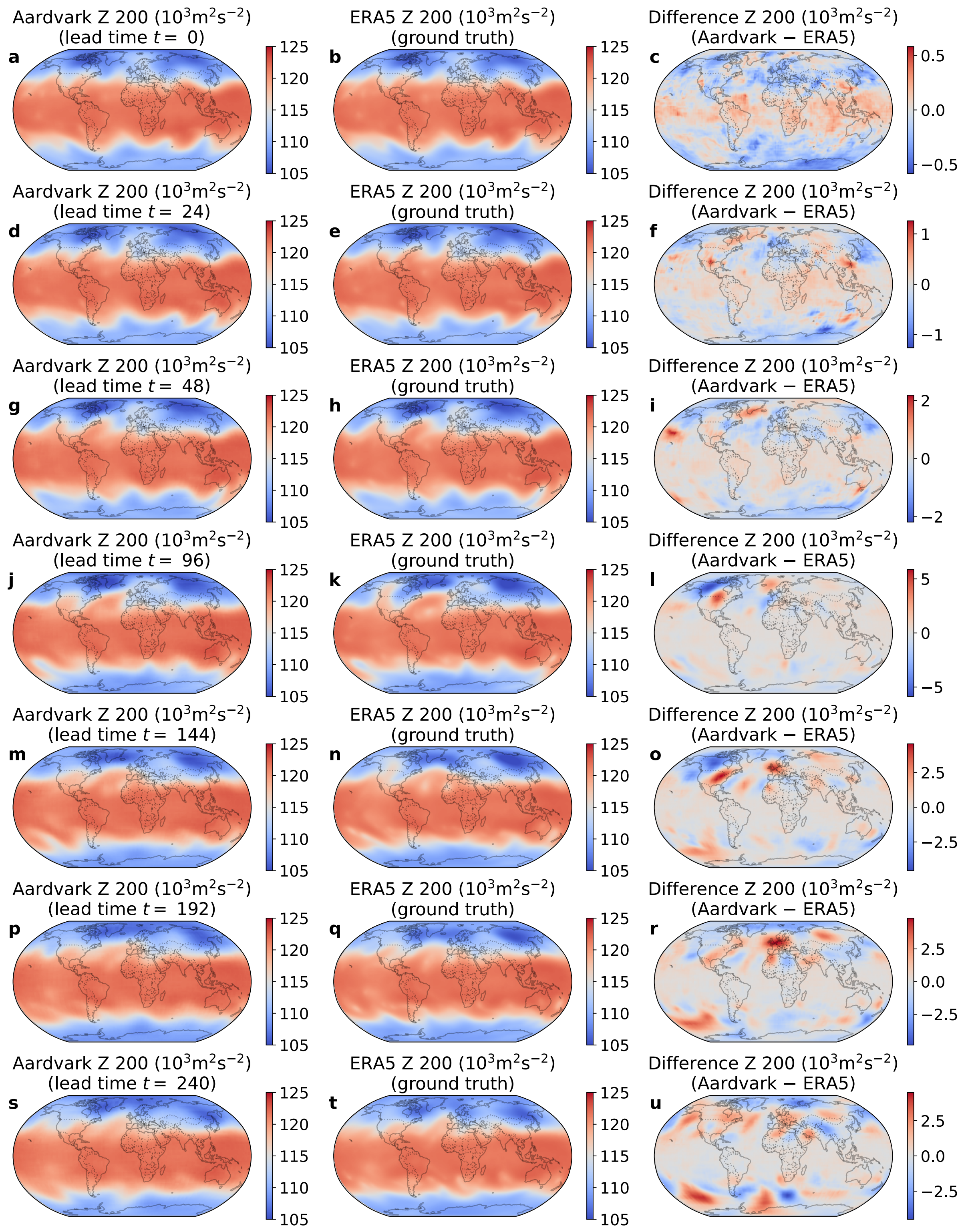

Figure 10. Illustration of Aardvark's forecasts against ERA5 [34]. *Note t = 0 is 11th January 2018.*

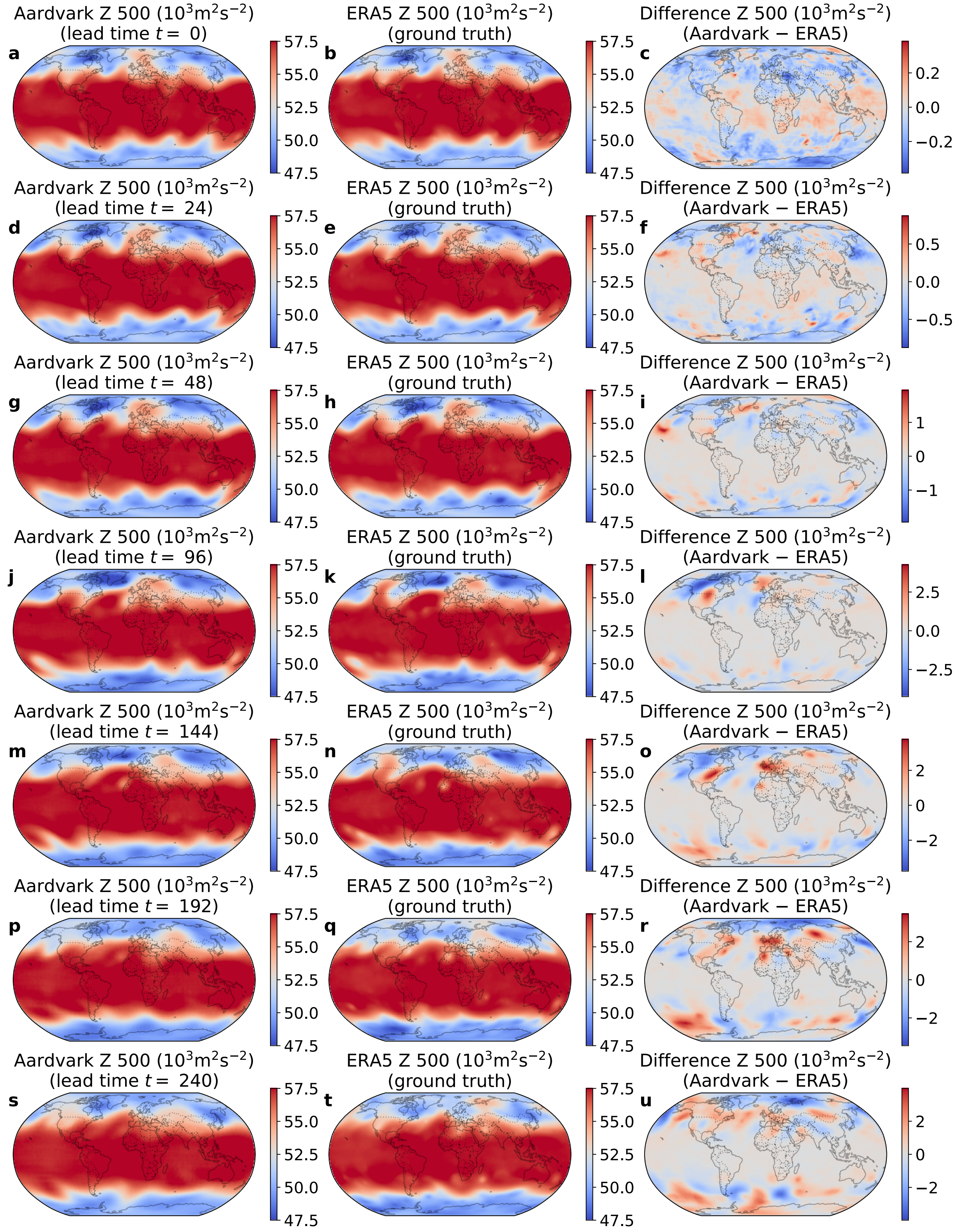

Figure 11. Illustration of Aardvark's forecasts against ERA5 [34]***.*** *Note t = 0 is 11th January 2018.*

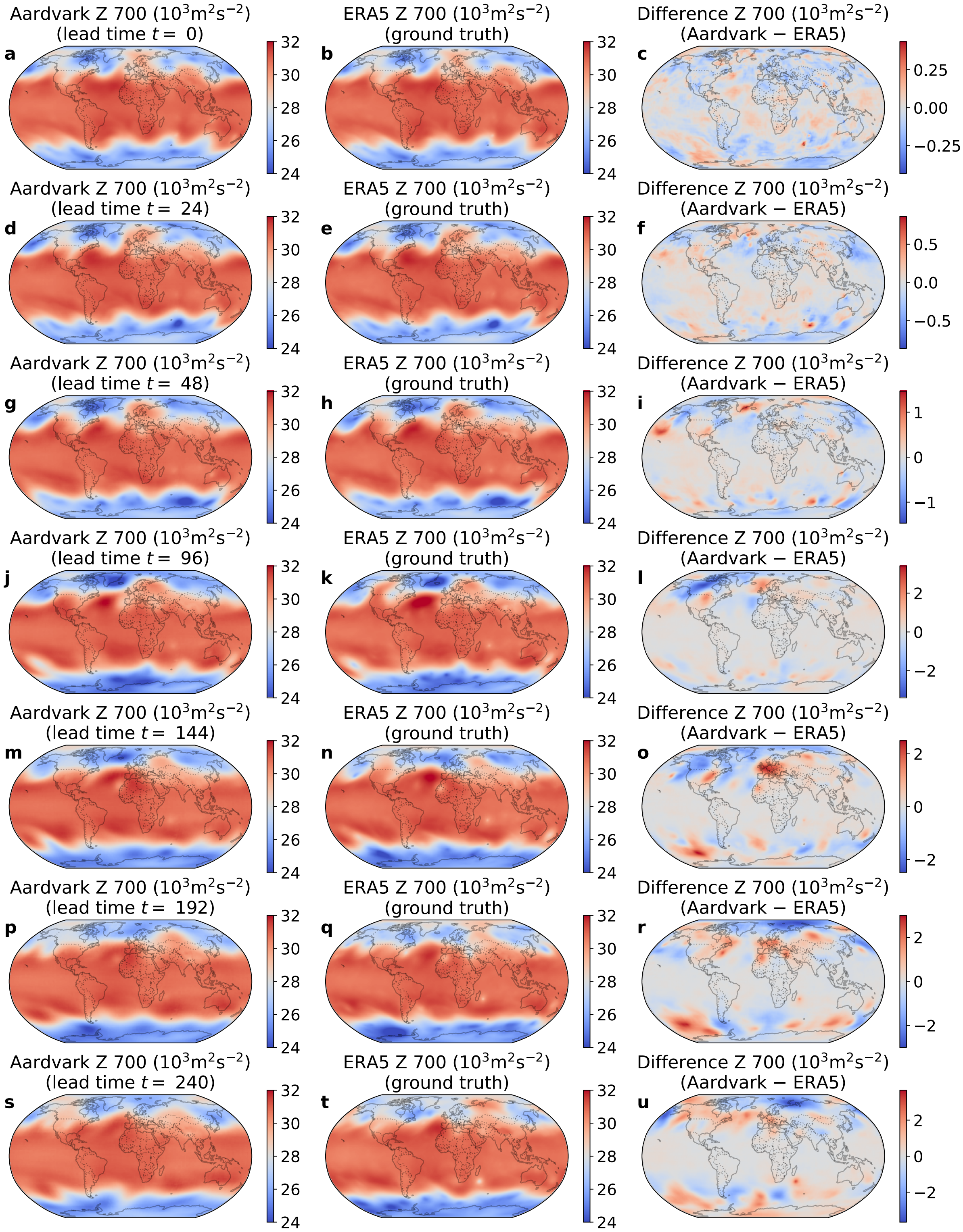

Figure 12. Illustration of Aardvark's forecasts against ERA5 [34]***.*** *Note t = 0 is 11th January 2018.*

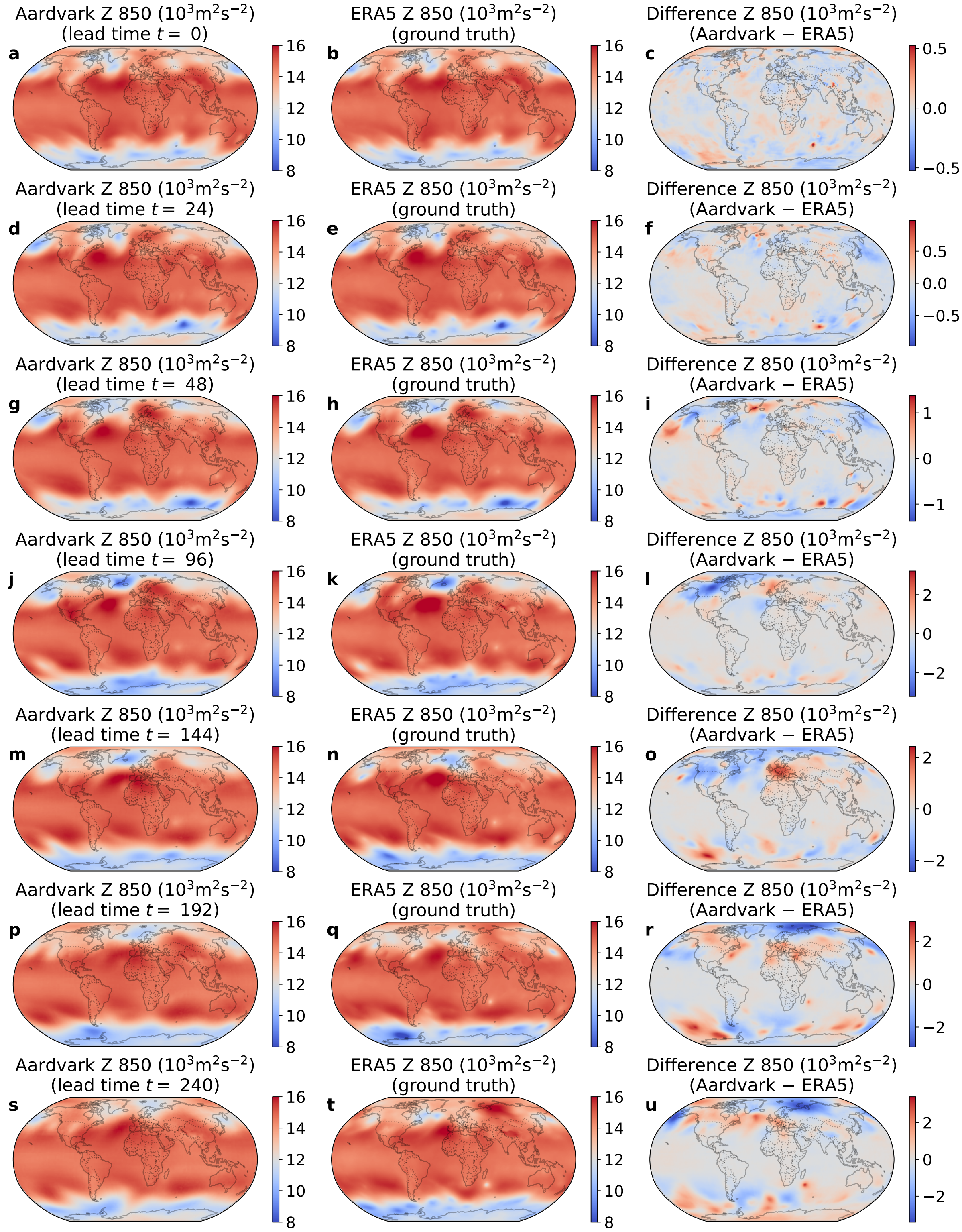

Figure 13. Illustration of Aardvark's forecasts against ERA5 **[34]*****.*** *Note t = 0 is 11th January 2018.*

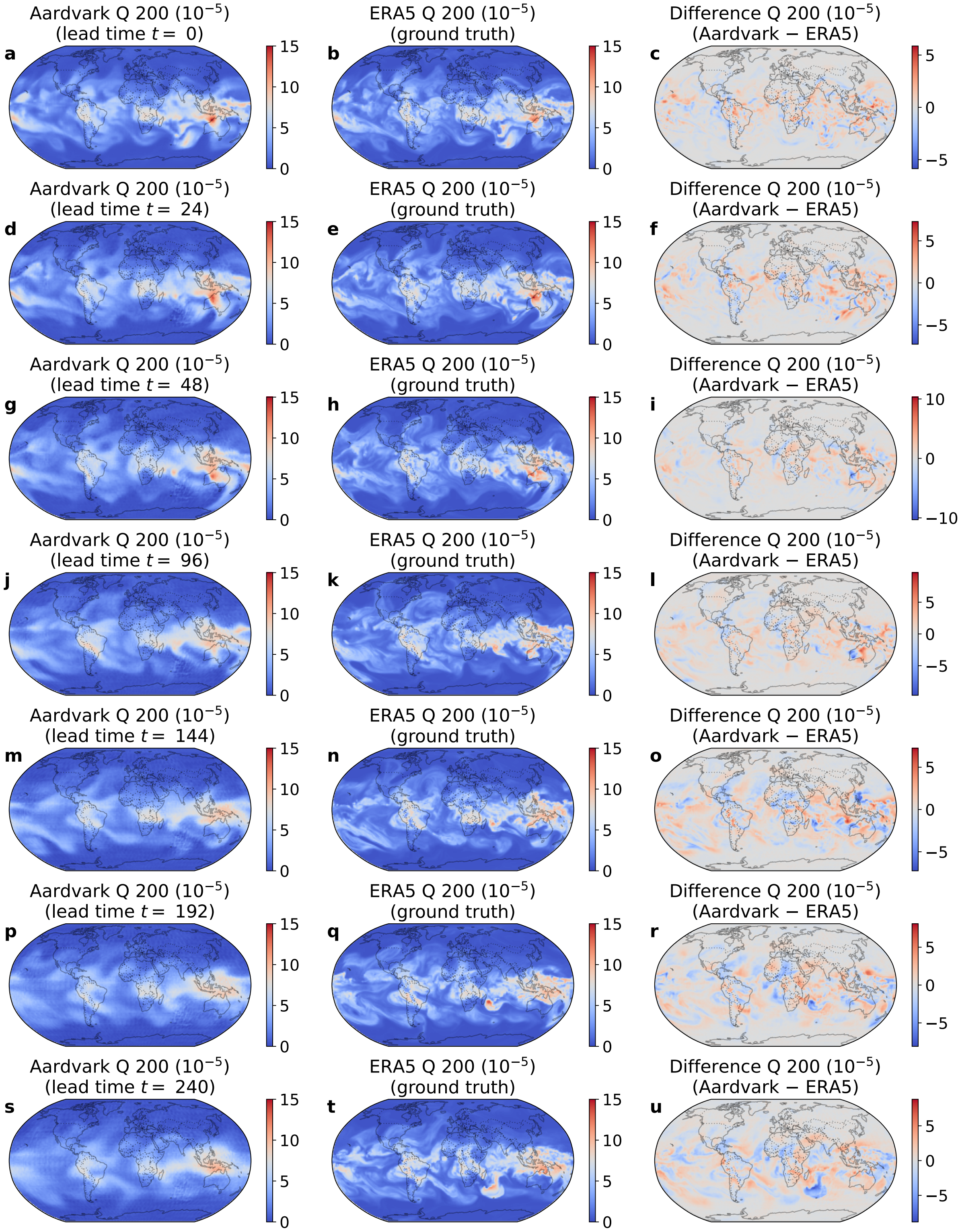

Figure 14. Illustration of Aardvark's forecasts against ERA5 [34]. *Note t = 0 is 11th January 2018.*

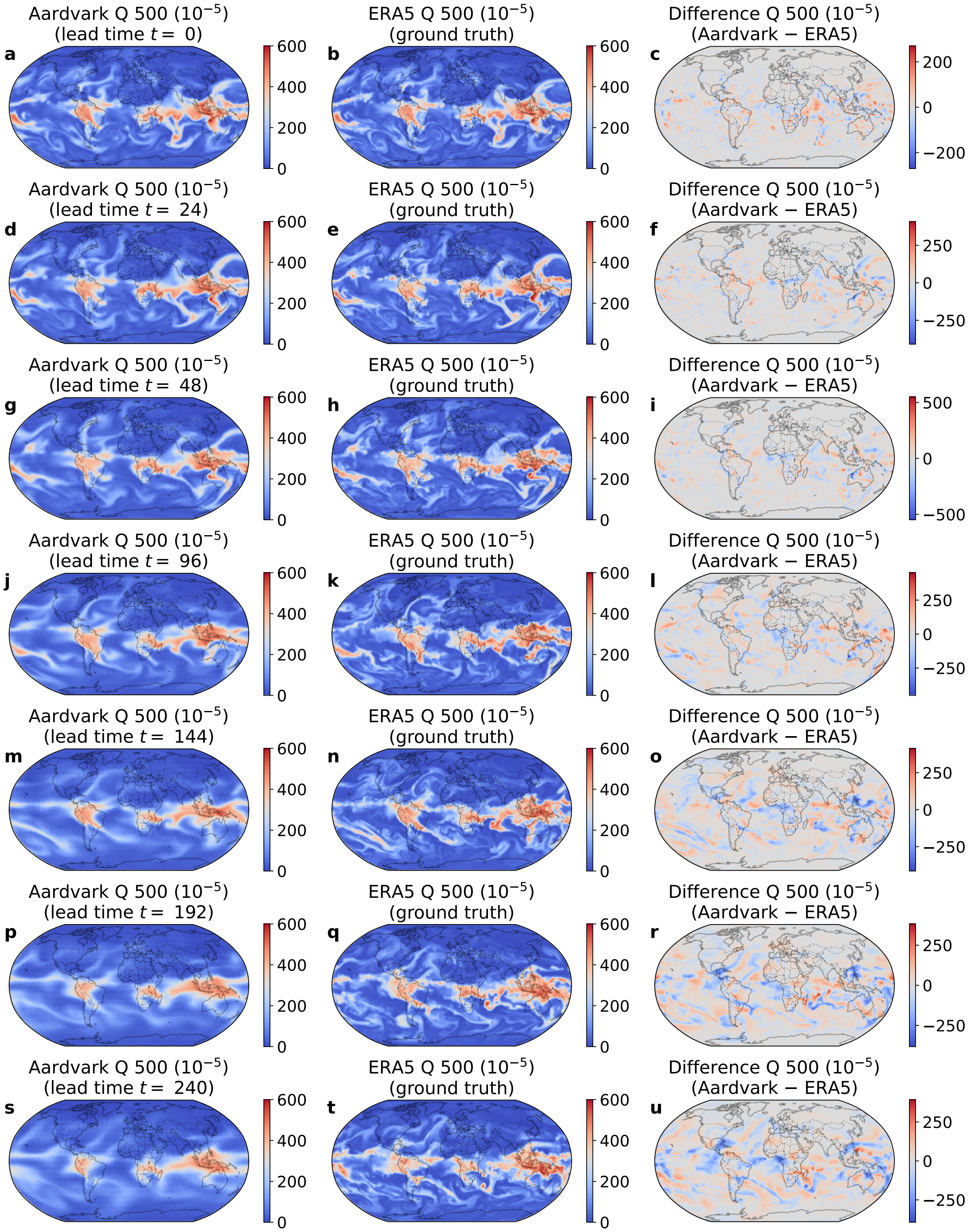

Figure 15. Illustration of Aardvark's forecasts against ERA5 [34]. *Note $t = 0$ is 11th January 2018.*

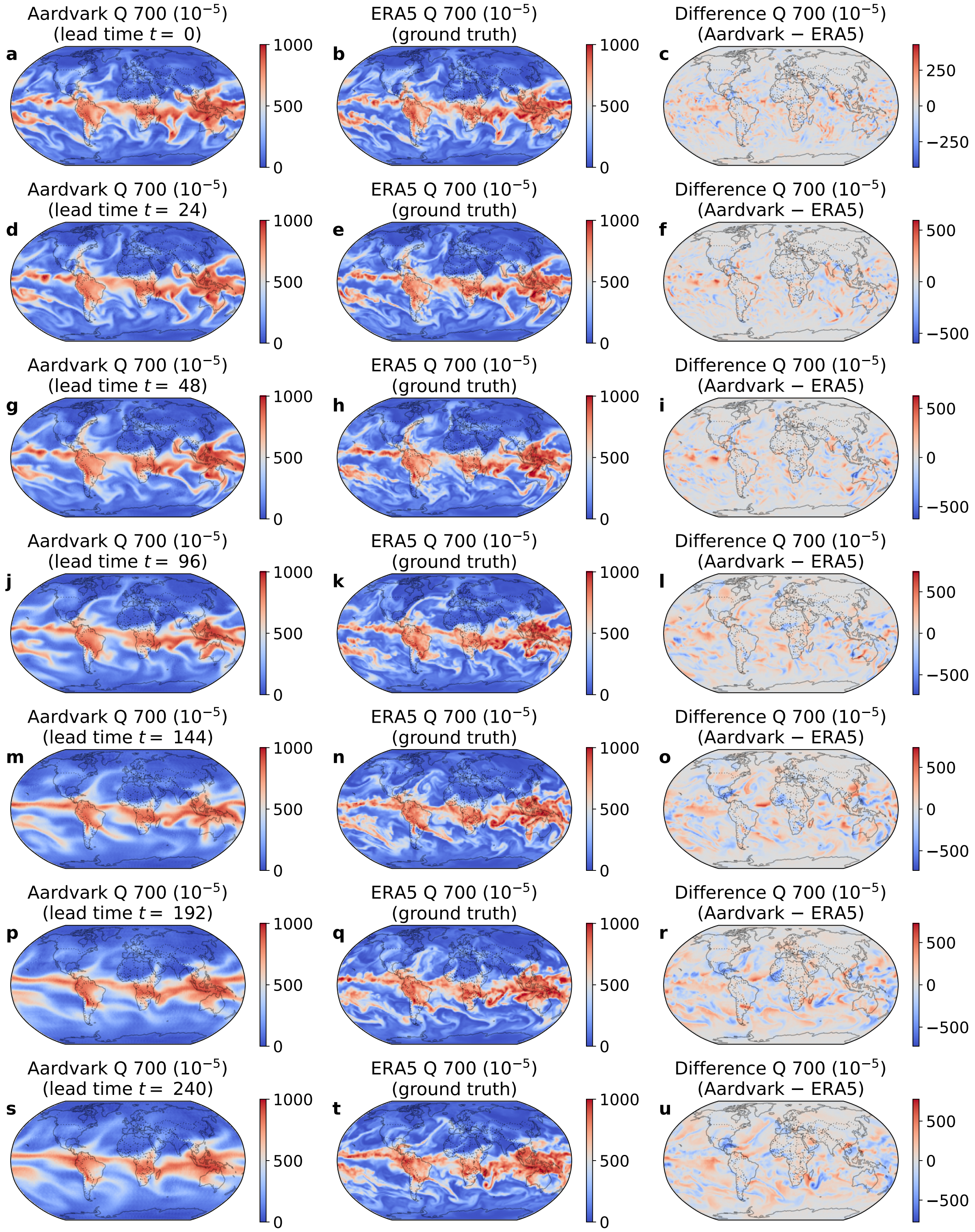

Figure 16. Illustration of Aardvark's forecasts against ERA5 [34]***.*** *Note t = 0 is 11th January 2018.*

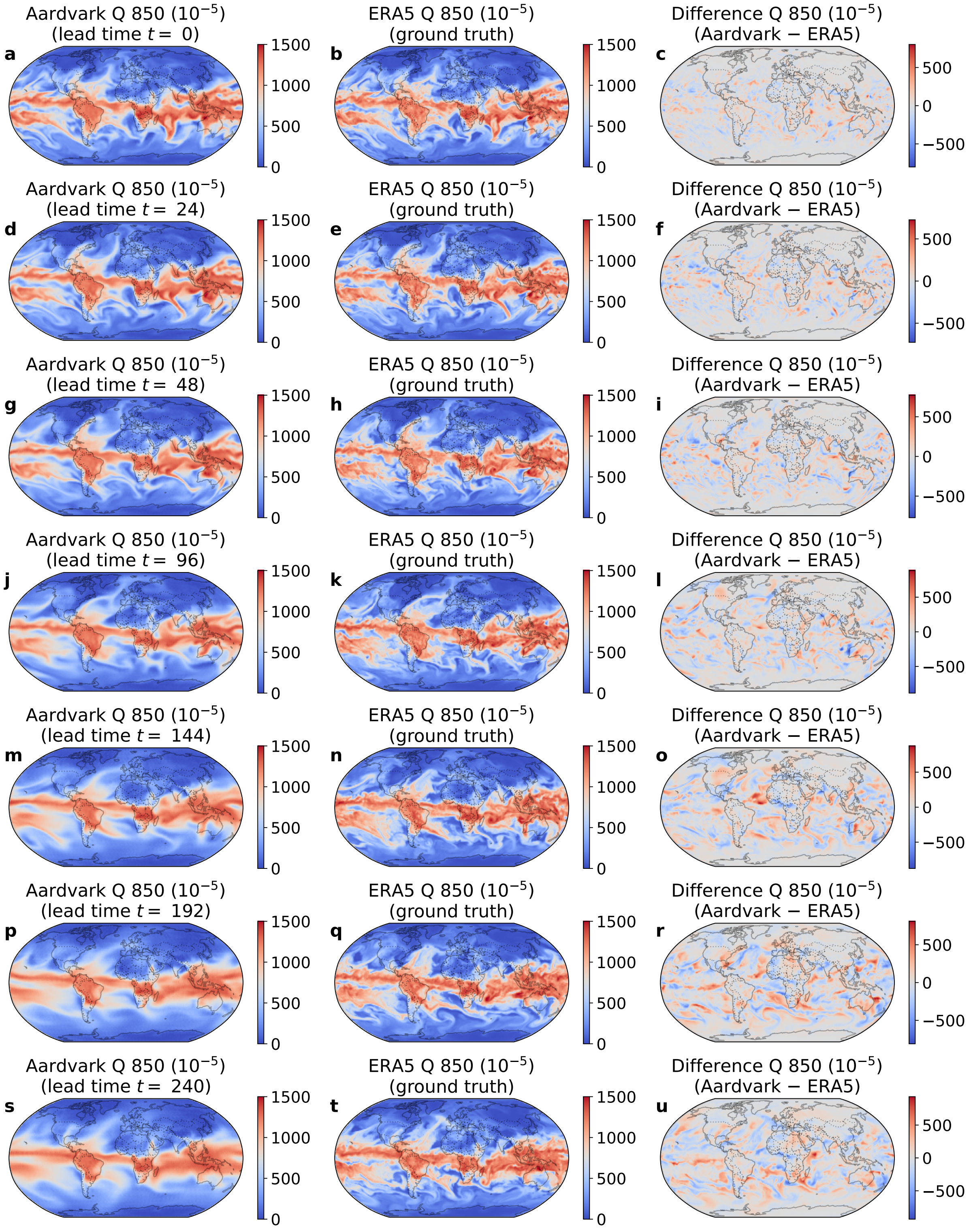

Figure 17. Illustration of Aardvark's forecasts against ERA5 [34]***.*** *Note t = 0 is 11th January 2018.*

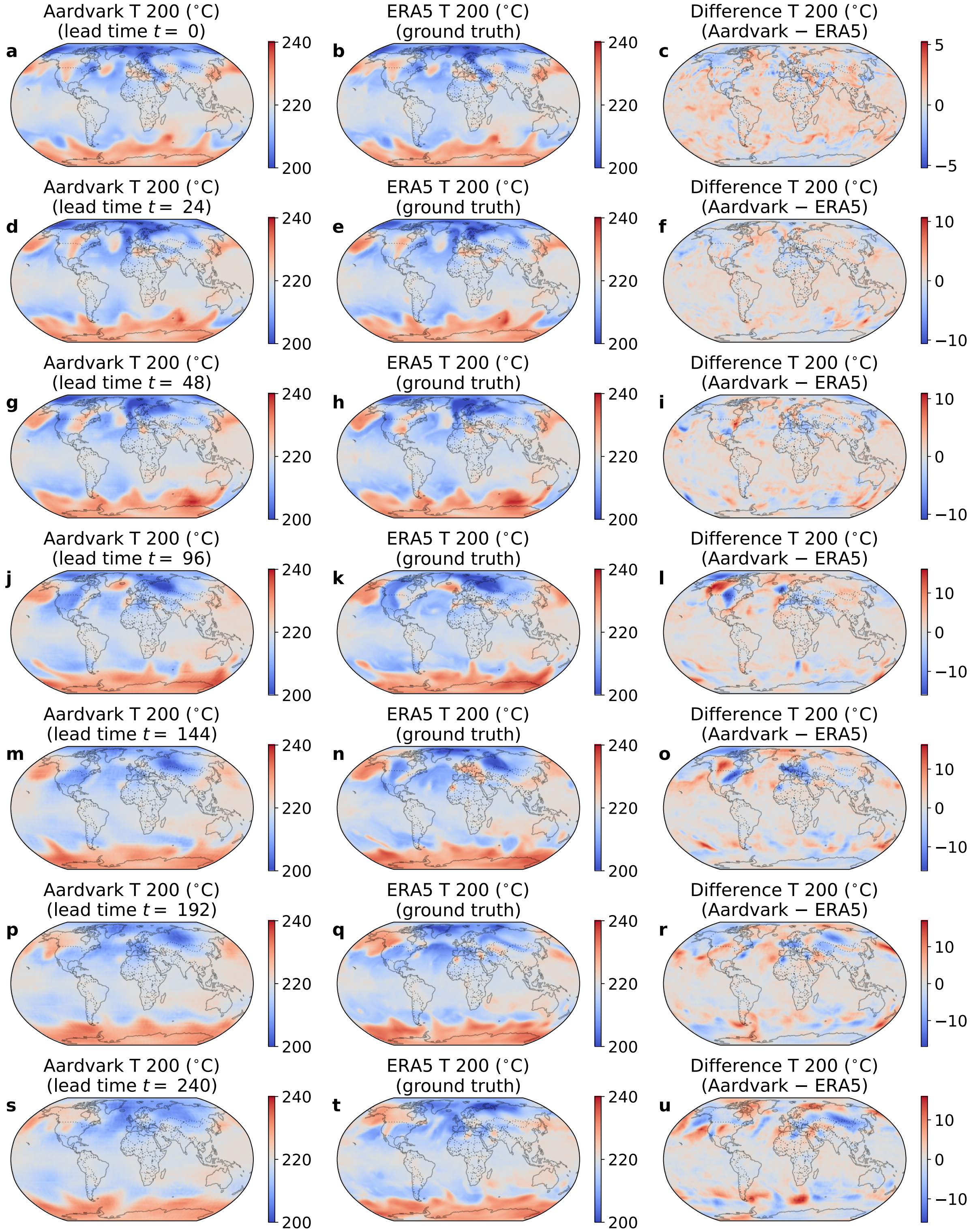

Figure 18. Illustration of Aardvark's forecasts against ERA5 [34]. *Note $t = 0$ is 11th January 2018.*

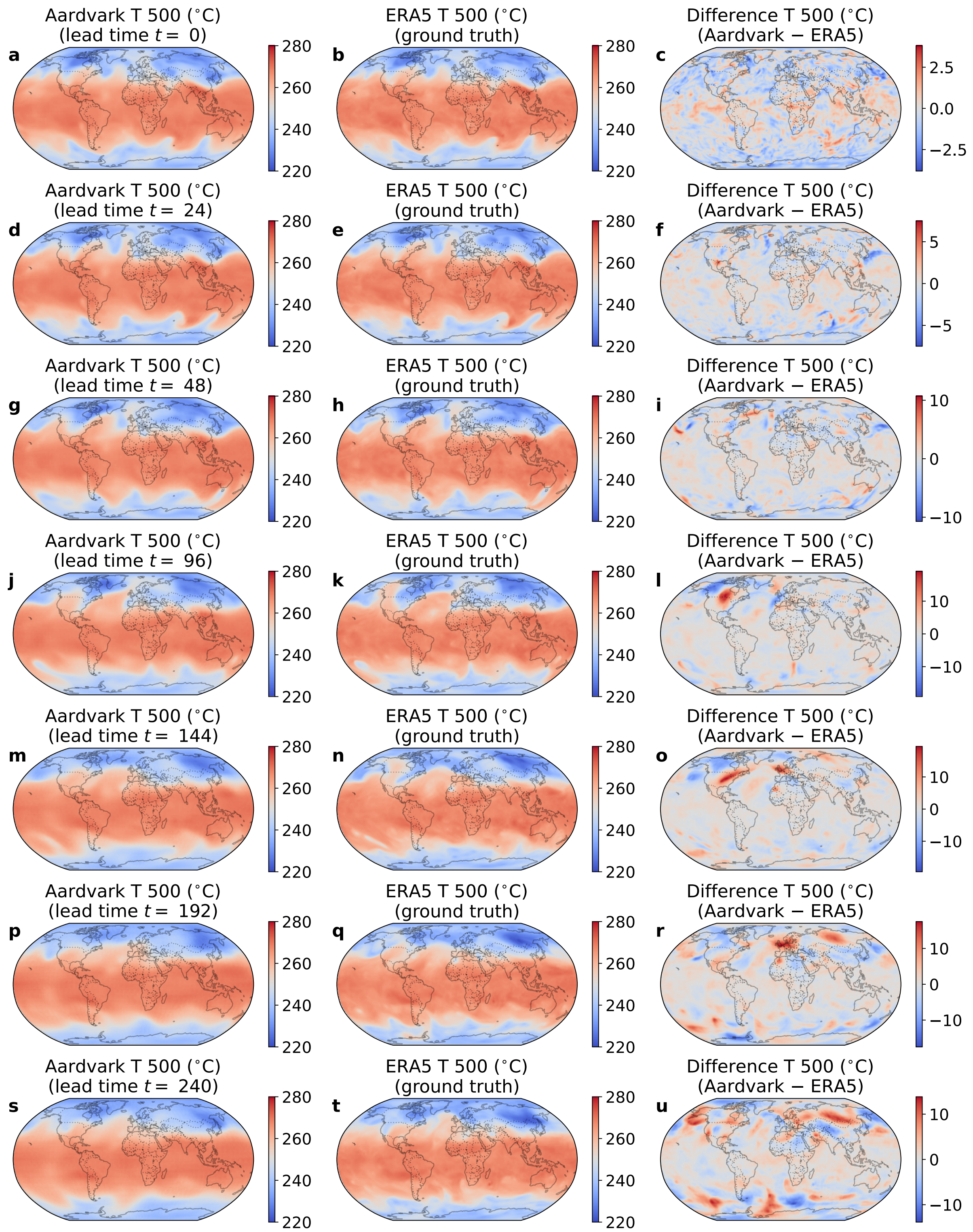

Figure 19. Illustration of Aardvark's forecasts against ERA5 [34]. *Note $t = 0$ is 11th January 2018.*

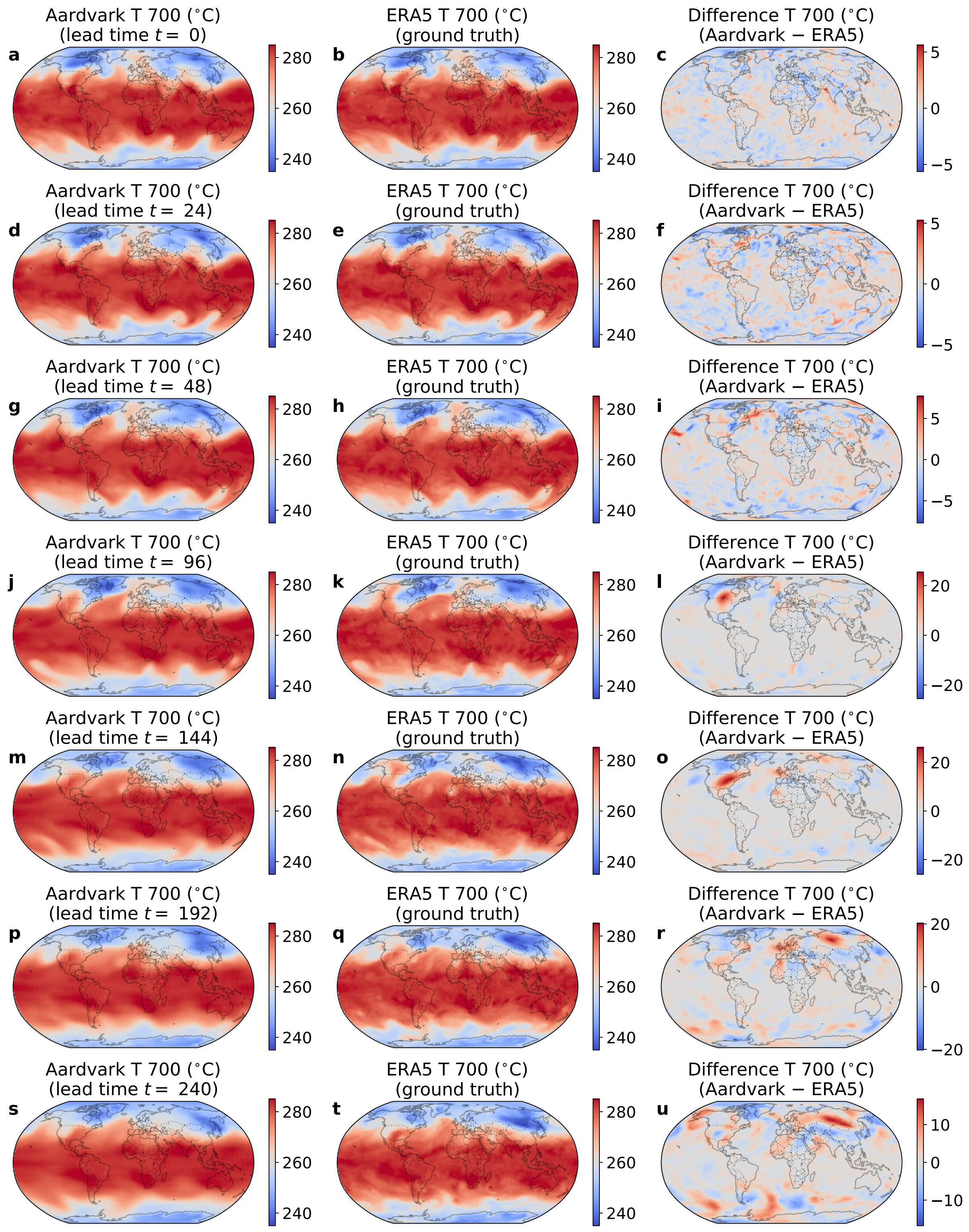

Figure 20. Illustration of Aardvark's forecasts against ERA5 [34]. *Note $t = 0$ is 11th January 2018.*

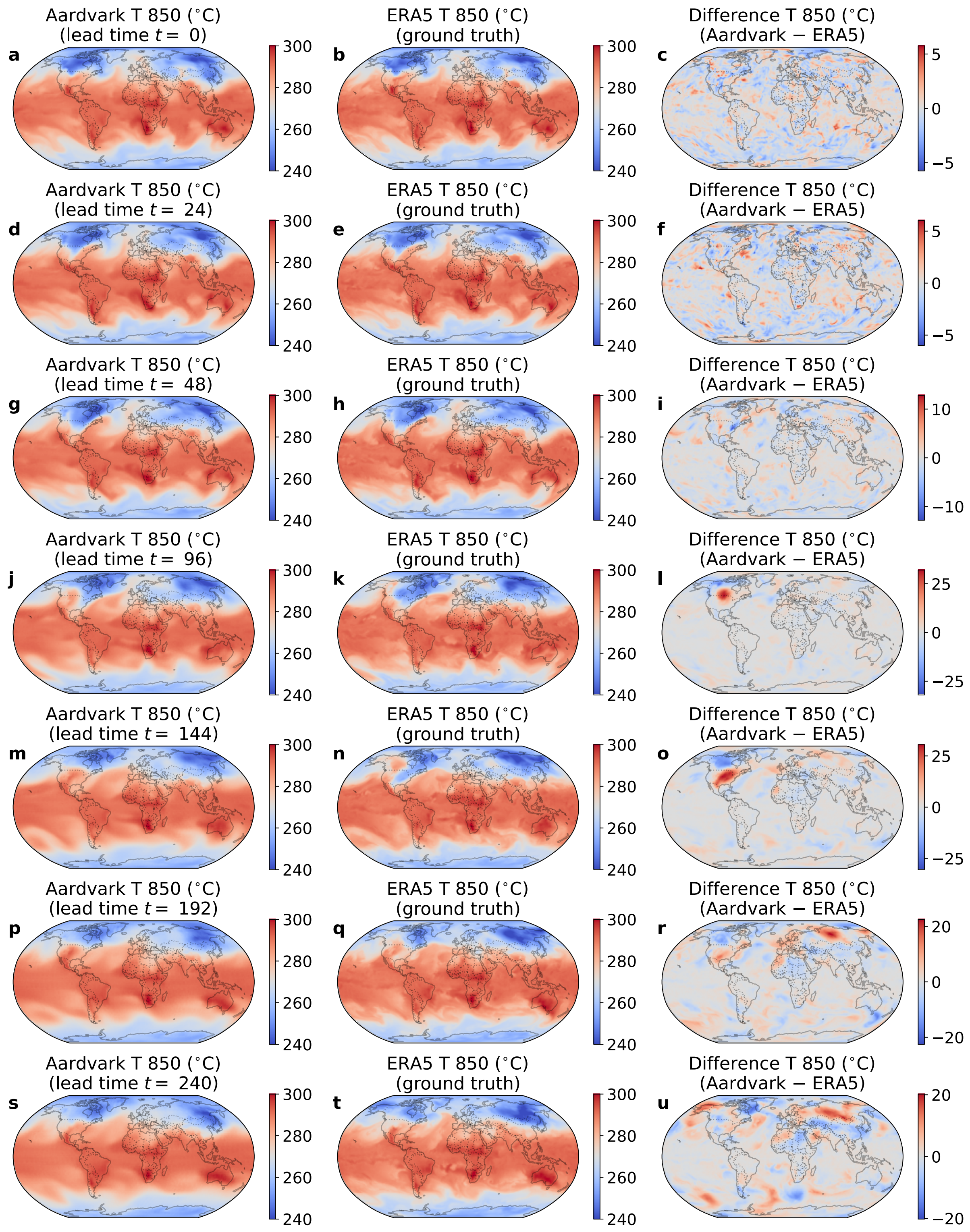

Figure 21. Illustration of Aardvark's forecasts against ERA5 [34]. *Note t = 0 is 11th January 2018.*

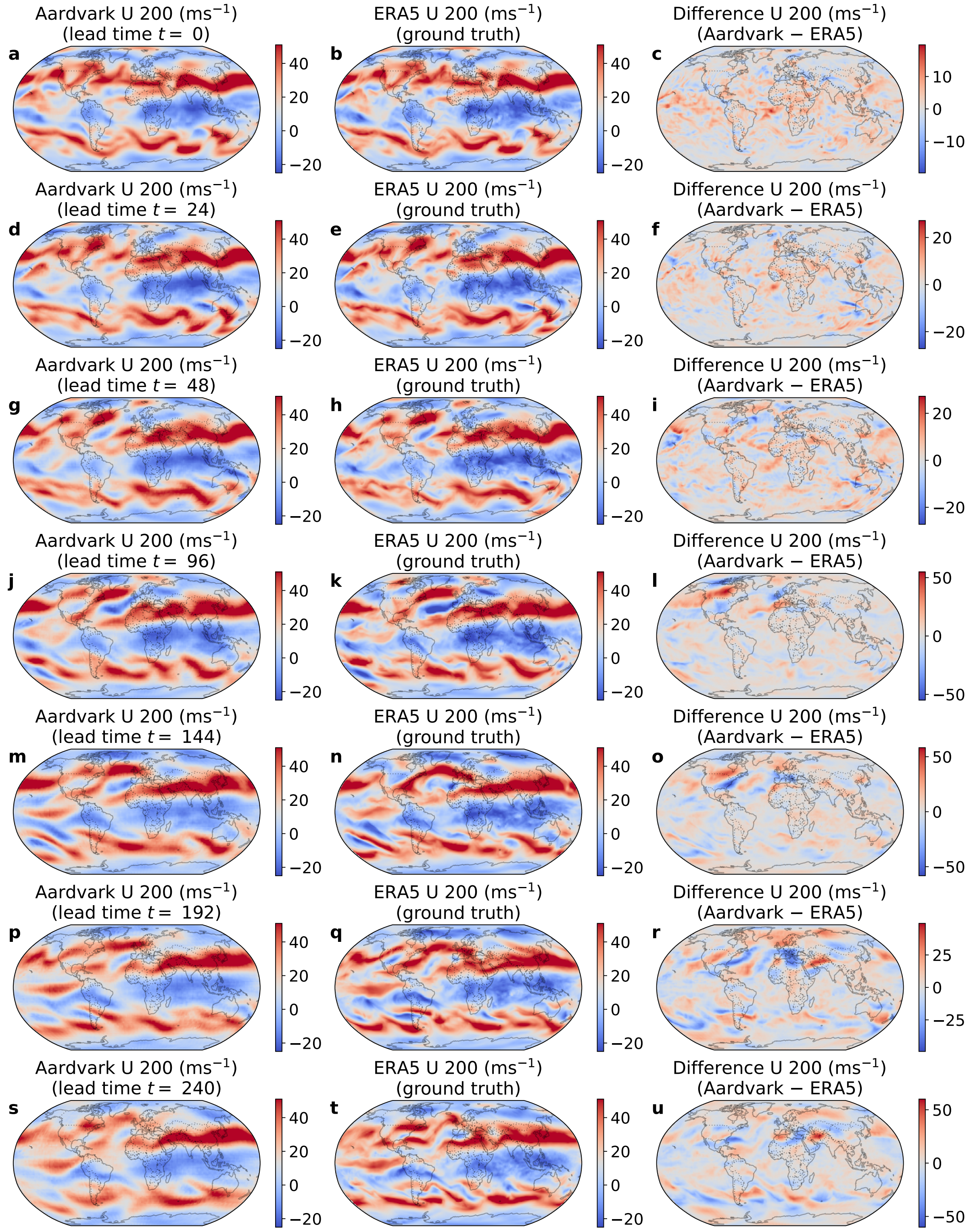

Figure 22. Illustration of Aardvark's forecasts against ERA5 [34]***.*** *Note t = 0 is 11*[th] *January 2018.*

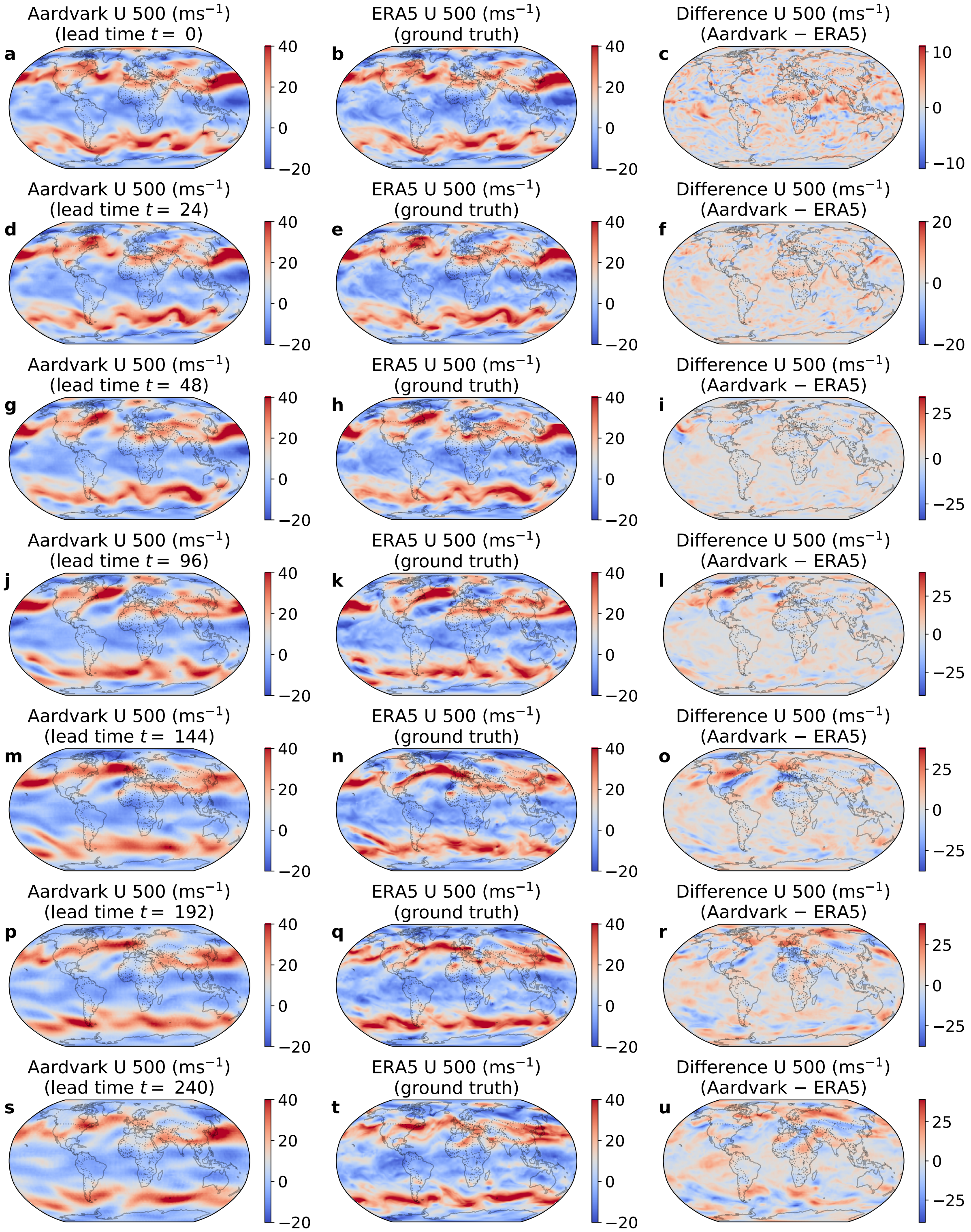

Figure 23. Illustration of Aardvark's forecasts against ERA5 [34]. *Note t = 0 is 11th January 2018.*

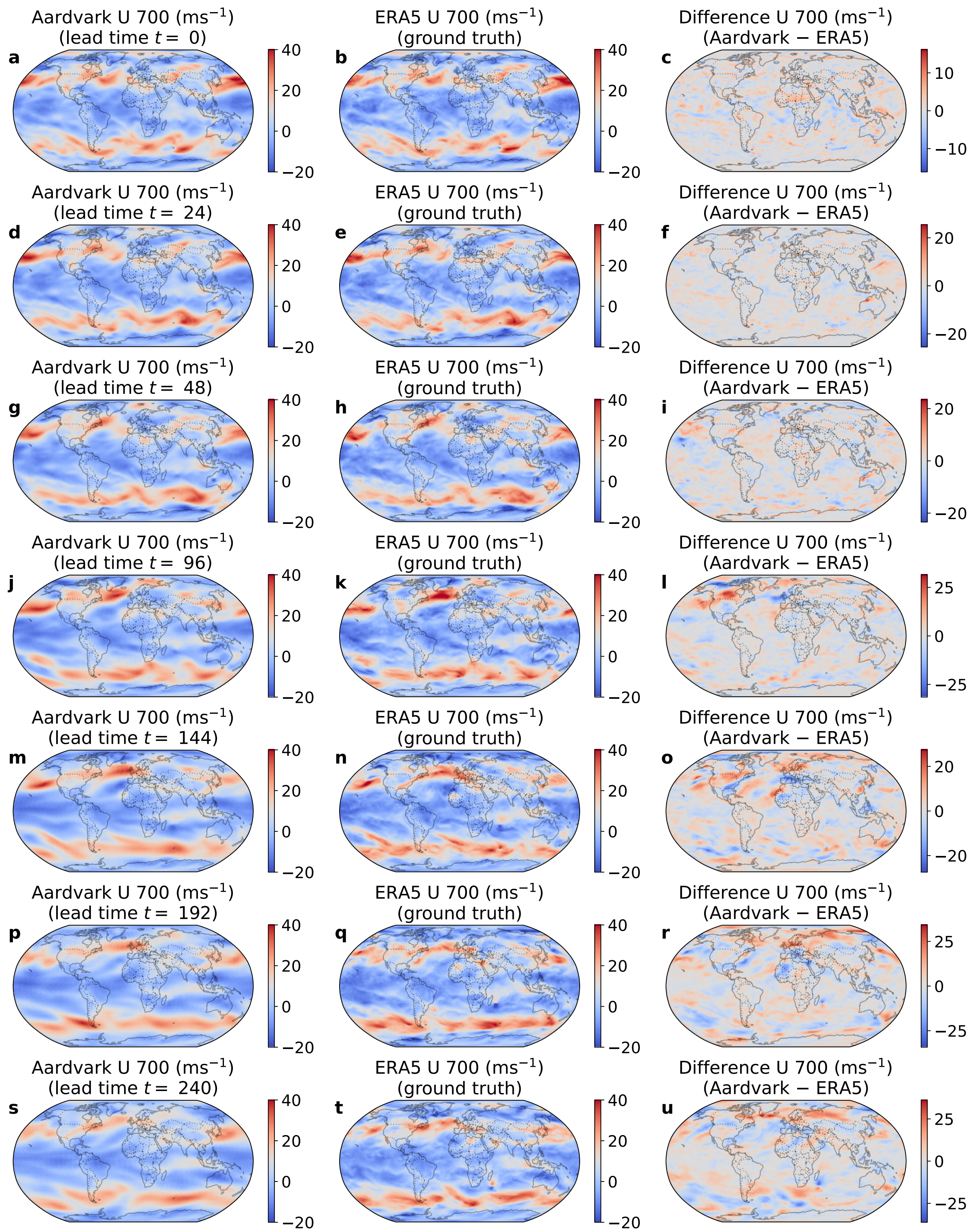

Figure 24. Illustration of Aardvark's forecasts against ERA5 [34]***.*** *Note t = 0 is 11th January 2018.*

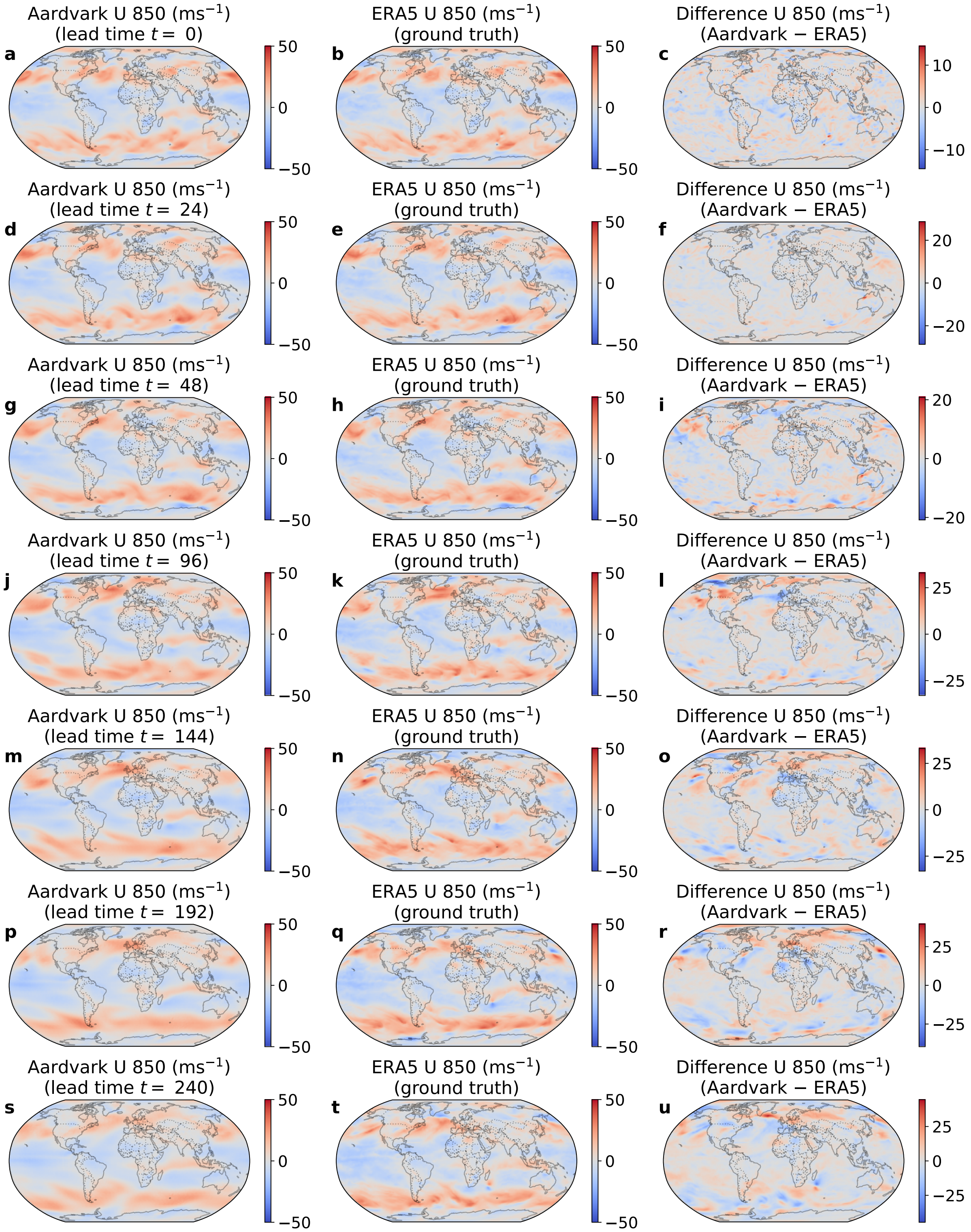

Figure 25. Illustration of Aardvark's forecasts against ERA5 [34]. *Note $t = 0$ is 11th January 2018.*

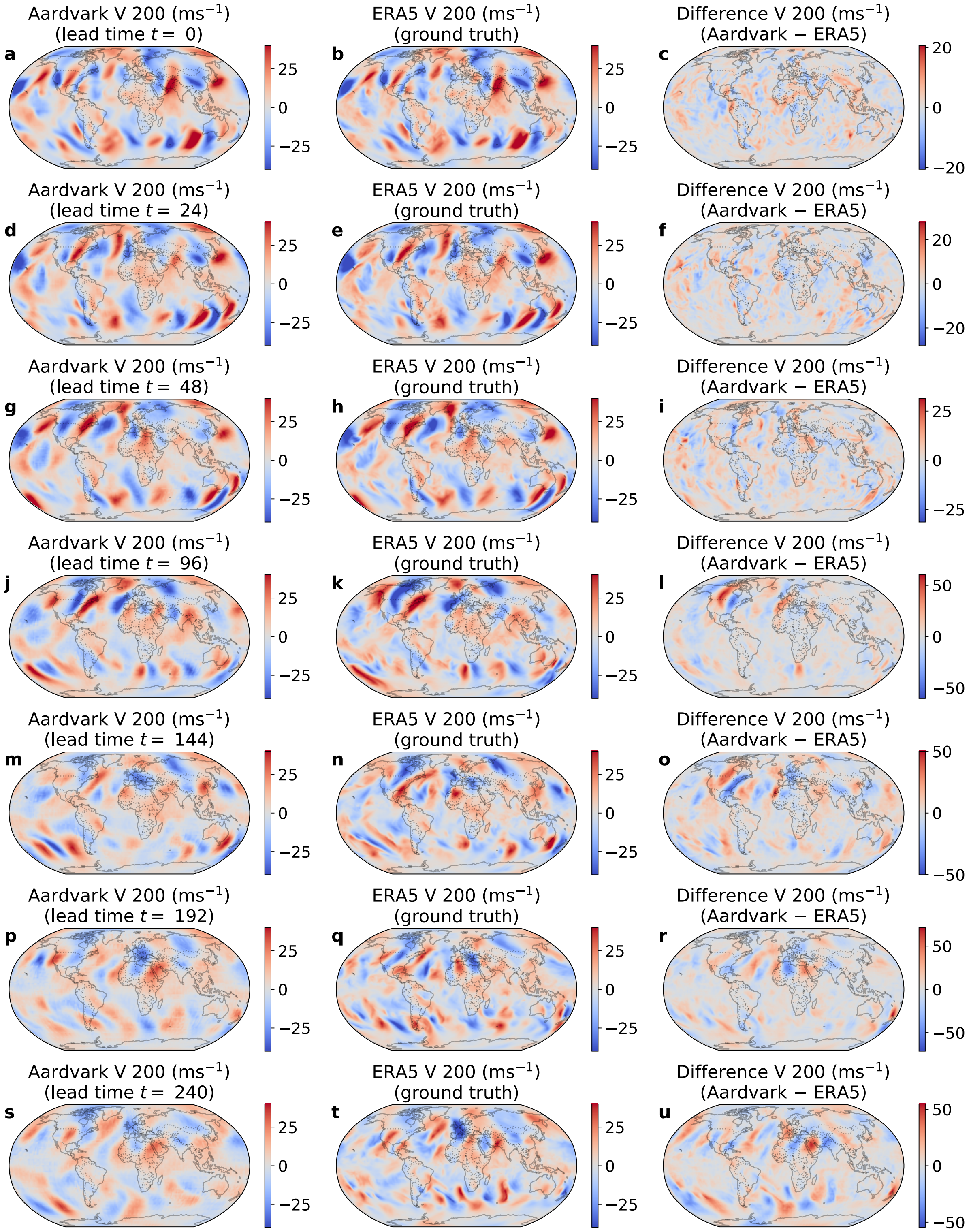

Figure 26. Illustration of Aardvark's forecasts against ERA5 [34]***.*** *Note $t = 0$ is 11th January 2018.*

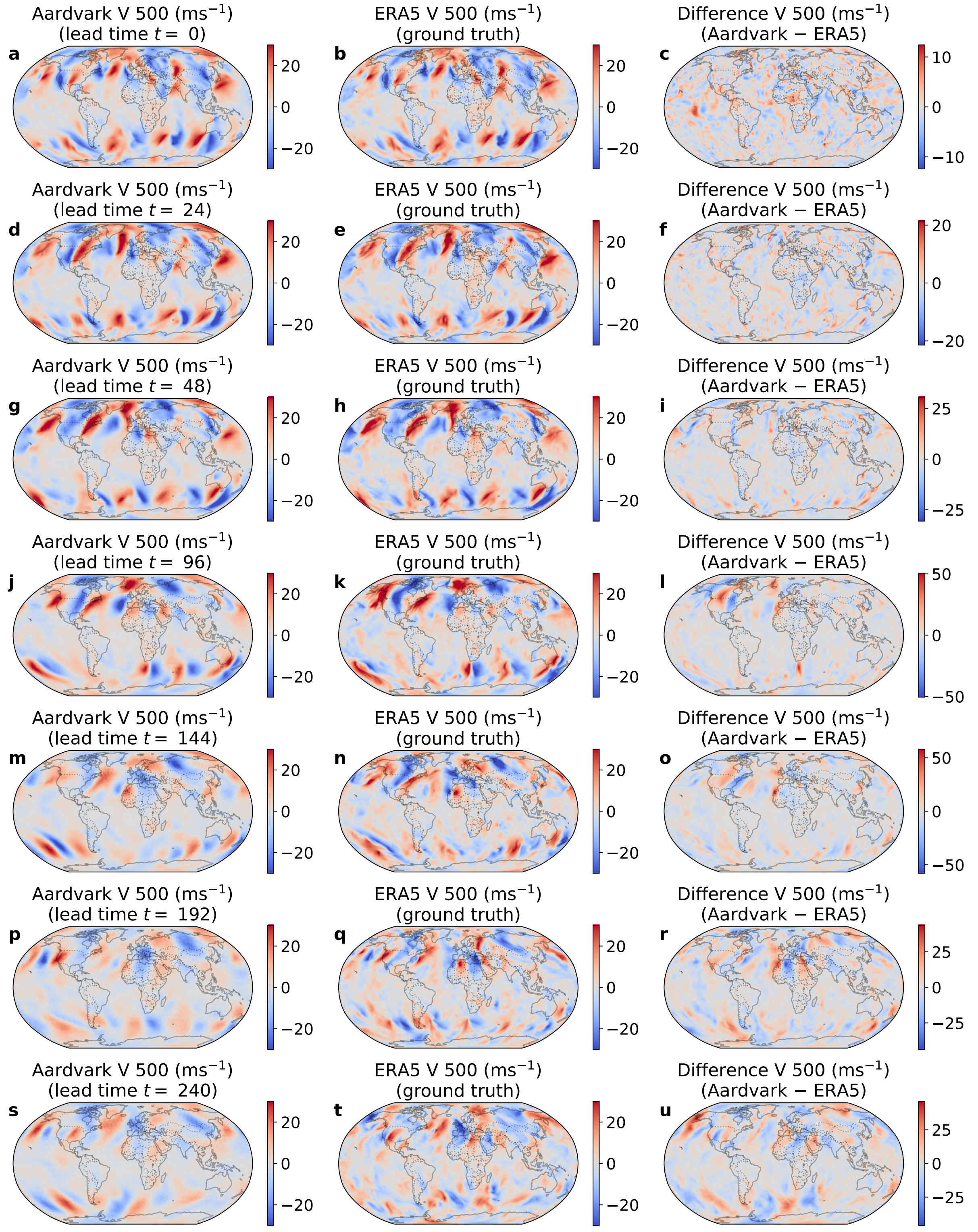

Figure 27. Illustration of Aardvark's forecasts against ERA5 [34]. *Note $t = 0$ is 11th January 2018.*

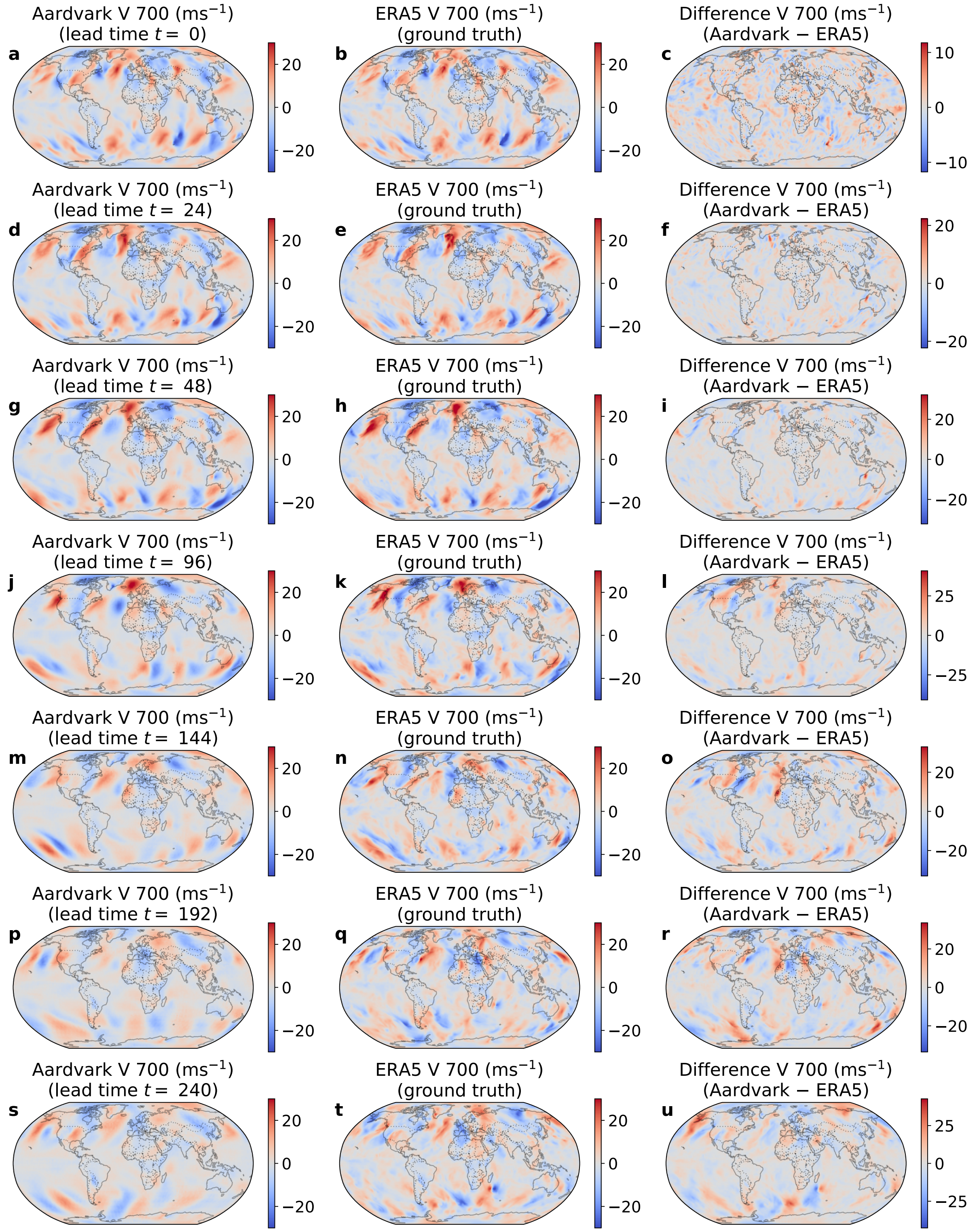

Figure 28. Illustration of Aardvark's forecasts against ERA5 [34]***.*** *Note t = 0 is 11th January 2018.*

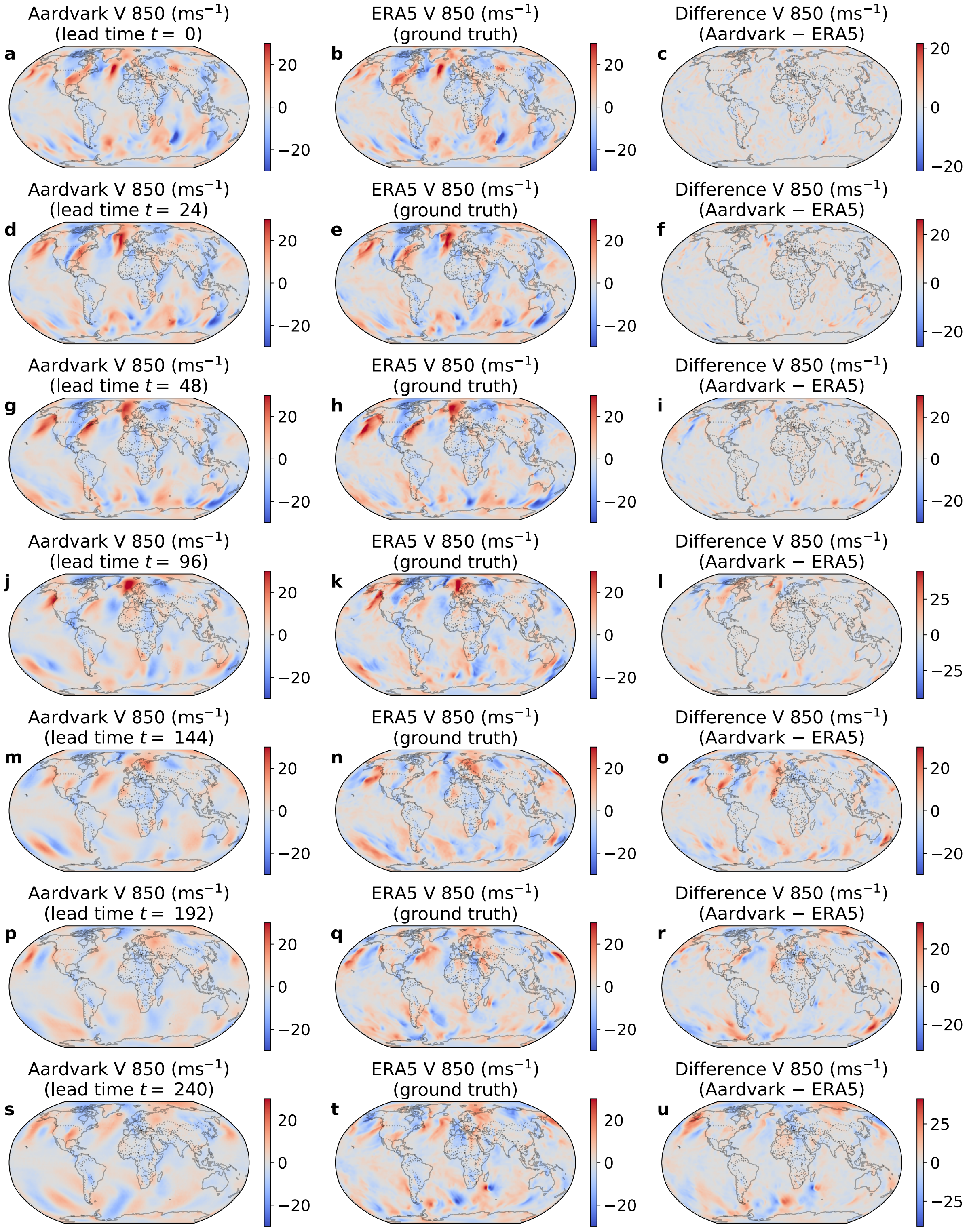

Figure 29. Illustration of Aardvark's forecasts against ERA5 [34]***.*** *Note t = 0 is 11th January 2018.*
